# Frequency of mixed neuropathologies in individuals with Down syndrome with and without Alzheimer's dementia

**DOI:** 10.1007/s00401-026-03028-z

**Published:** 2026-05-15

**Authors:** Lisi Flores-Aguilar, Thomas D. Zaikos, Isabel Rivera, Sierra T. Wright, Jerry Lou, Brianna Gawronski, Lourdes Gonzalez, Jillian V. Berry, Jeremy Rouanet, Natalie C. Edwards, Dan K. Hoang, Kevin Wood, Ann-Charlotte Granholm, Elliott J. Mufson, Edwin S. Monuki, Milos D. Ikonomovic, Julia Kofler, Eric W. Doran, Ira T. Lott, Minodora O. Totoiu, Christy L. Hom, Florence Lai, William H. Yong, Frederick A. Schmitt, Jordan Harp, Peter T. Nelson, Jose Gutierrez, Patrick J. Lao, Donna M. Wilcock, Adam M. Brickman, Elizabeth Head

**Affiliations:** 1 Department of Pathology and Laboratory Medicine, University of California Irvine, Irvine, CA 92697 USA; 2 Department of Microbiology and Molecular Genetics, University of California Irvine, Irvine, CA USA; 3 Institute for Memory Impairments and Neurological Disorders (MIND), University of California Irvine, Irvine, CA USA; 4 Department of Neurology, University of California Irvine, Irvine, CA USA; 5 Taub Institute for Research On Alzheimer’s Disease and the Aging Brain, Columbia University, New York, NY USA; 6 Department of Neurology, Vagelos College of Physicians and Surgeons, Columbia University, New York, NY USA; 7 Department of Neuroscience, Columbia University, New York, NY USA; 8 Department of Neurosurgery, University of Colorado Anschutz Medical Center, Aurora, CO USA; 9 Department of Translational Neuroscience and Neurology, Barrow Neurological Institute, Phoenix, AZ USA; 10 Sue and Bill Gross Stem Cell Center, University of California Irvine, Irvine, CA USA; 11 Department of Neurology, University of Pittsburgh, Pittsburgh, PA USA; 12 Department of Psychiatry, University of Pittsburgh, Pittsburgh, PA USA; 13 Geriatric Research Education and Clinical Center, VA Pittsburgh HS, Pittsburgh, PA USA; 14 Department of Pathology, University of Pittsburgh, Pittsburgh, PA USA; 15 Department of Pediatrics, University of California Irvine, Orange, CA USA; 16 Department of Psychiatry and Human Behavior, University of California Irvine, Irvine, CA USA; 17 Department of Neurology, Harvard University, Cambridge, MA USA; 18 Department of Neurology, Sanders-Brown Center On Aging, University of Kentucky, Lexington, KY USA; 19 Department of Pathology and Laboratory Medicine, Division of Neuropathology, University of Kentucky, Lexington, KY USA; 20 Department of Neurology, Indiana University, Bloomington, IN USA

**Keywords:** Trisomy 21, Cerebral amyloid angiopathy, TDP-43, Lewy pathology, Resilience and resistance to AD

## Abstract

Individuals with Down syndrome (DS) develop Alzheimer's disease neuropathological change (ADNC) by the age of 40 years, and most develop dementia by their early 50s. The frequency of co-pathologies in clinically and neuropathologically characterized adults with DS has not been systematically characterized. We characterized the frequency of ADNC and common co-pathologies, including cerebral amyloid angiopathy (CAA), Lewy pathology (LP), limbic predominant age-related TDP-43 encephalopathy neuropathological change (LATE-NC), hippocampal sclerosis (HS), and other cerebrovascular and macroscopic findings reported in standardized National Alzheimer’s Coordinating Center (NACC) neuropathology forms in 63 adults with DS over 40 years. A secondary exploratory objective was to compare the neuropathological profiles between individuals with (n = 55) and without (n = 8) dementia from the same autopsy cohort. In the full autopsy cohort, cortical and hippocampal atrophy, and moderate-to-severe locus coeruleus hypopigmentation was a common finding. Pure ADNC, was present in only 29% of individuals. CAA was the most frequent co-pathology, present in approximately 84% of individuals followed by LP (21%), HS (19%), and LATE-NC (17%). Atherosclerosis and arteriolosclerosis were infrequent. In exploratory comparisons between dementia groups, brain weight was significantly lower in individuals with dementia than in those without (900 ± 116 vs 1060 ± 108 g P = .0006), and severe hippocampal atrophy and locus coeruleus hypopigmentation were more frequent in those with dementia (P = .049, P = .009, respectively). Advanced Braak NFT stage, frequent neuritic plaques, and high ADNC were more frequent in individuals with dementia (P = .0001, P = .03, P = .0016, respectively). LATE-NC and HS occurred exclusively in individuals with dementia, while LP and CAA were found in both groups. Individuals without dementia showed a less complex co-pathology profile than those with dementia. Our findings demonstrate that co-pathologies are present in people with DS, and that despite their genetic predisposition to AD, some individuals with DS may exhibit resilience and resistance mechanisms to AD.

**Supplementary Information:**

The online version contains supplementary material available at 10.1007/s00401-026-03028-z.

## Introduction

Down syndrome (DS) is caused by the triplication of chromosome 21 and is the most common genetic form of intellectual disability. Trisomy of chromosome 21 leads to neurodevelopmental deficits resulting in reduced brain size, cerebellar hypoplasia, reduced hippocampal volume, narrowness of the superior temporal gyrus, larger subcortical gray matter volumes, cortical dysgenesis, reduced number of neurons, among others [11, 22, 34, 35, 54, 62, 91, 92, 113, 123, 126]. Given that the Amyloid Precursor Protein (*APP*) gene is encoded in chromosome 21, nearly all individuals with DS have a lifelong overproduction of amyloid-beta (Aβ), and develop a full-blown AD pathology in a predictable pattern consistent with Thal and Braak NFT staging by 40 years of age [25, 68]. Dementia typically presents in their early-to-mid 50s, with a reported range of 40 to 70 years [46]. Importantly, premorbid intellectual disability level has not been associated with variability in the trajectory of AD biomarkers or the timing of cognitive decline in DS, suggesting that neurodevelopmental differences in DS do not impact AD associated cognitive trajectories [39]. Further, rare cases of partial trisomy of chromosome 21, lacking triplication of the *APP* gene, suggest that overexpression of this protein is necessary to develop ADNC and dementia [28, 93]. Notably, while ADNC is nearly universal in adults with DS, there is heterogeneity in the onset of dementia, and a small subset of individuals remains cognitively stable throughout their lifespans [14]. This suggests the existence of individuals with DS who are resilient to cognitive decline and the possibility of being resistant to developing AD neuropathology. This observation has given rise to the recent definition of resilience and resistance to AD in DS [14]. Resilience refers to better-than-expected cognitive performance relative to adults with DS with similar ADNC and intellectual disability while resistance refers to the absence or reduction of AD pathology compared to other adults with DS within the same age range [14]. So far, the neuropathological profile of these individuals has not been described, despite its relevance for providing insights into protective mechanisms against cognitive decline in this population.

Alzheimer’s disease neuropathologic change, defined by the accumulation of amyloid plaques and neurofibrillary tangles (NFTs), is frequently accompanied by additional pathologies such as cerebral amyloid angiopathy (CAA), Lewy pathology (LP), limbic predominant age-related TDP-43 encephalopathy neuropathological change (LATE-NC), hippocampal sclerosis (HS), and cerebrovascular disease (atherosclerosis, arteriolosclerosis, infarcts, hemorrhages) with the presence of these co-pathologies lowering the threshold for dementia [50, 100–102, 110, 115]. Therefore, in most cases, ADNC does not occur in the absence of other co-pathologies, and dementia may be the result of the convergence and possible interaction of multiple co-pathologies rather than a single disease process. Most of these co-pathologies have been studied in large autopsy cohorts of aging individuals and late-onset AD (LOAD); however, small autopsy studies have characterized the frequency of co-pathologies in DS. Such studies report that CAA, LP, LATE-NC and HS are present in adults with DS [19, 24, 25, 41, 44, 64, 65, 123]. However, these studies have been limited by small sample sizes and lack of systematic neuropathological and clinical assessment methods. Such limitations underscore the need for larger, well-characterized cohorts to better characterize the occurrence of co-pathologies in DS.

The primary objective of this study was to characterize the frequency and spectrum of ADNC and common co-pathologies, including CAA, LP, LATE-NC, HS, and other cerebrovascular features, in a well clinically and neuropathologically characterized autopsy cohort of adults with DS from the University of California ADRC (UCI-ADRC), the University of Kentucky ADRC (UKY-ADRC) and the Alzheimer Biomarker Consortium–Down Syndrome (ABC-DS) by using standardized National Alzheimer’s Coordinating Center (NACC) forms with systematic and harmonized data collection across sites. A secondary and exploratory objective was to describe the neuropathological profile of a rare subset of individuals with DS who remained cognitively stable until death and to compare it with that of those that developed dementia. We hypothesized that, similar to patterns observed in the aging and AD population, frequency of co-pathologies would be common in adults with DS. We further hypothesized that individuals with DS without dementia, would display a distinct neuropathological profile compared to those with dementia. Given the small number of individuals with DS without dementia in our autopsy cohort, the comparisons between dementia groups are considered exploratory; yet we deem these cases warrant further description as they may provide early insights into the factors underlying possible resilience and resistance mechanisms to AD in DS.

## Methods

**Fig.1:**
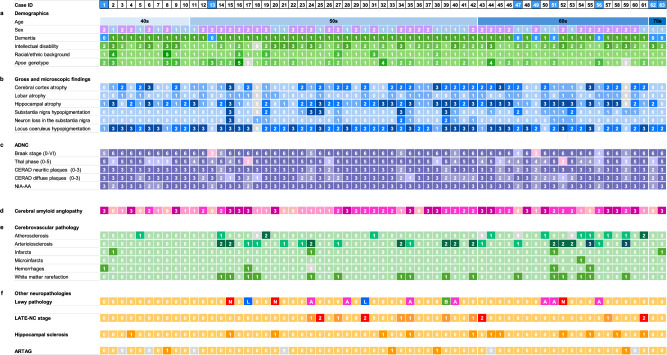
Demographical, clinical, and neuropathological data from DS cases. **a** Demographics: *Sex* = males (1), females (2); *dementia status* = without dementia (0), with dementia (1), case IDs of cases without dementia are highlighted in blue; *intellectual disability* = mild (1), moderate (2), severe/profound (3); *race/ethnicity* = white (1), Native Hawaiian or Pacific islander (2), Hispanic/latino (3), Asian (4), Black/African American (5), *APOE genotype*: ε3/ε3 (1), ε3/ε4 (2), ε2/ε3 (3), ε4/ε4 (4), ε2/ε4 (5). **b** Gross findings: *Atrophy of the cerebral cortex and hippocampus, hypopigmentation of the substantia nigra and locus coeruleus and neuronal loss in the substantia nigra* = none (0), mild (1), moderate (2), severe (3). *Lobar atrophy* = absent (0), present (1). **c** ADNC: *Braak NFT stage 0-VI* = (0—6); *Thal phase* = (0—5); *CERAD scores* = (0—3); *NIA-AA* = intermediate ADNC (2), high ADNC (3). **d** Cerebral amyloid angiopathy: none (0), mild (1), moderate (2), severe (3). **e** Cerebrovascular pathology: *atherosclerosis, arteriolosclerosis* = none (0), mild (1), moderate (2), severe (3); *microinfarcts, hemorrhages, white matter rarefaction* = absent (0), present (1). **f** Other neuropathologies: *Lewy pathology* = absent (0), amygdala predominant (A), brainstem-predominant (B), limbic (L), neocortical (N). *LATE-NC* = absent (0), present (1), LATE-NC stage 1 (1), LATE-NC stage 2 (2); *hippocampal sclerosis and ARTAG* = absent (0), present (1). 9 = missing data. APOE = Apolipoprotein E, ADNC = Alzheimer’s disease neuropathologic change, NIA-AA = National Institute on Aging-Alzheimer’s Association, NFT = neurofibrillary tangle, CERAD = Consortium to Establish a Registry for Alzheimer’s disease, TDP-43 = TDP: Transactive response DNA-binding protein 43 kDa (TDP-43), LATE-NC = Limbic-predominant age-related TDP-43 encephalopathy neuropathological change, ARTAG = Aging-related tau astrogliopathy

Neuropathology reports from the autopsy cohorts of the University of California Irvine (UCI) and University of Kentucky (UKY)-Alzheimer Disease Research Centers (ADRCs), and the Alzheimer Biomarker Consortium – Down Syndrome study (ABC-DS) study from 2002 to 2023, were reviewed. Demographic data are presented in Fig. 1 and Table 1. Data from 63 individuals with DS with and without a diagnosis of dementia aged 40 years and older were captured using the NACC neuropathology forms (versions 7–9, *n* = 27; versions 10–11, *n* = 36). Thirty-two cases were included in a previous study of cerebrovascular pathology frequency in DS [41]. Of note, the earlier study did not assess the presence of co-pathologies in the DS brain and only investigated cerebrovascular pathology in individuals with DS with dementia. Race, ethnicity, and sex were reported by the donor's family and categorized according to NIH classification standards.

**Table 1:** Demographics of the study

Characteristic	All DS n (%)	DS without dementia n (%)	DS with dementia n (%)	P value
Age	63	8	55	
Mean (SD), years	56 ± 6.2	60 ± 9	56 ± 6	0.07
Range	42–70	42–70	43–66	
Sex	63	8	55	
Females	25 (40)	4 (50)	34 (62)	0.70
Intellectual disability	62	8	54	
Mild	20	3	17	.71
Moderate	26	4	22	
Severe/Profound	16	1	15	
PMI	61	8	53	
Median (IQR), hours	5 (4–7)	5 (4–7)	5 (4–8)	.97
Range	2–27	3–15	2–27	
Race	63	8	55	
African American	1 (2)	0	1 (2)	> .99
Asian	1 (2)	0	1 (2)	
Hispanic/Latino	4 (6)	0	4 (7)	
Native Hawaiian or Pacific Islander	1 (2)	0	1 (2)	
White	56 (89)	8 (100)	48 (87)	
*APOE* genotype	61	8	53	
ε3/ε3	36 (59)	4 (50)	32 (60)	.45
ε3/ε4	17 (28)	2 (25)	15 (28)	
ε2/ε3	5 (8)	2 (25)	3 (6)	
ε4/ε4	2 (3)	0	2 (4)	
ε2/ε4	1 (2)	0	1 (2)	

*SD* standard deviation *PMI* postmortem interval, *IQR* interquartile range, *APOE* Apoliporotein E

The first column presents findings for the full DS autopsy cohort (All DS). The following columns present findings stratified by dementia status and group comparisons

Postmortem interval estimates the time between death and tissue procurement. Some percentages do not total 100 due to rounding

### Neuropathological evaluation

Neuropathological evaluations were performed by board-certified neuropathologists who were blinded to the clinical status (TDZ, WHY, MDI, JL, PTN). For this study, we included the following findings:

*Gross findings:* Following gross visual inspection, the presence and severity of atrophy and hypopigmentation were assessed semi-quantitatively. Atrophy of the cerebral cortex and hippocampus was assessed as none, mild, moderate, or severe and lobar atrophy (significant frontal and/or temporal atrophy) was recorded as present or not present. Since these measures were not included in versions 7–9, hippocampal and lobar atrophy descriptions were reviewed on neuropathological reports. The hippocampal descriptors included in these reports were: “small/quite small”, “very small” or “strikingly small”. To harmonize data across versions, these descriptors were converted to mild, moderate or severe atrophy, respectively. Hypopigmentation of the substantia nigra (SN) and locus coeruleus (LC) were assessed grossly and neuronal loss in the SN by looking at hematoxylin and eosin (H&E) stainings. The severity of hypopigmentation and neuronal loss were scored as none, mild, moderate, or severe.

*AD neuropathology:* Braak staging was used to evaluate the distribution and severity of tau pathology (Total tau, Agilent #A0024, 1:3000) [15]. Amyloid pathology, including cerebral amyloid angiopathy (CAA), was assessed by amyloid-beta (Aβ) immunostaining (6E10, Biolegend #803,015, 1:1000). The Consortium to Establish a Registry for Alzheimer’s disease (CERAD) criteria were used to assess neuritic and diffuse Aβ plaques [75, 76]. Thal staging was used to evaluate the anatomical location of Aβ and was scored in versions 10–11 [1, 76, 117].The NIA-Alzheimer’s Association (AA) ADNC guidelines were used to assess based on three neuropathological hallmarks, amyloid plaques (A), Braak NFT stage (B), and neuritic plaque score (C). Using this ABC scoring rubric, the model assigns the overall ADNC severity level as low, intermediate, or high. Intermediate or high ADNC is sufficient for a neuropathologic diagnosis of dementia due to AD [76].

*Cerebrovascular pathology:* Guidelines to evaluate CAA severity were adapted from [87, 121]. CAA burden on parenchymal and/or leptomeningeal vessels was semi-quantitatively assessed by scoring the overall severity as none, mild, moderate, and severe. Atherosclerosis was evaluated in the circle of Willis by gross visual inspection as described by Beach et al. [10]. The obstruction of the arteries was rated based on severity in the following manner: none, mild (30–50% obstruction), moderate (50–70% obstruction), or severe (> 70% obstruction). Arteriolosclerosis or concentric hyaline thickening of the media of arterioles (40–150 μm in diameter) associated with concentric stenosis of the vessel lumen, was assessed by H&E staining in the subcortical white or gray matter of the middle frontal gyrus, superior and middle temporal gyrus, inferior parietal and occipital cortices, basal ganglia, amygdala, cingulate cortex and hippocampus. The severity of hyalinosis of the media and adventitia of parenchymal and/or leptomeningeal vessels was scored as none, mild, moderate, or severe. White matter rarefaction or white matter pallor was assessed as present or absent in the centrum semiovale and subcortical white matter. Brain mineralization of blood vessels was scored as present or absent. There were some differences between forms 7–9 and 10–11 when assessing infarcts, microinfarcts, and hemorrhages. The main difference is that recent forms (versions 10–11) differentiate between old/chronic and acute infarcts, microinfarcts, and hemorrhages. Further, the most recent versions differentiate microbleeds from gross hemorrhages. For this study, data were harmonized, and acute and old/chronic infarcts were grouped into one category, and the same was done for acute and old/chronic microinfarcts. Hemorrhages and microbleeds were also grouped into one category. The presence of infarcts and hemorrhages was assessed in the cerebral cortex, basal ganglia, amygdala, hippocampus, brainstem, and cerebellum.

*LP:* A non-phospho-specific ⍺-synuclein polyclonal antibody (Milipore Sigma #AB5038, 1:1000) was used to detect LP (i.e., Lewy bodies and Lewy neurites), which was characterized following the LP diagnostic criteria [5]. This criterion applies a dichotomous approach (absent vs. present) to score LP and includes the following categories: olfactory-only, amygdala predominant, brainstem, limbic, and neocortical LP. As olfactory bulb pathology was inconsistently reported, we did not evaluate for this category of LP for this study.

*TDP-43 pathology:* A non-phospho-specific TDP-43 polyclonal antibody (Proteintech, #10,782-2-AP, 1:2000) was used to detect TDP-43 pathology in the amygdala, mid-level hippocampus, and middle frontal gyrus. LATE-NC staging was done following established guidelines [84]. Stage 1 corresponds to TDP-43 pathology in the amygdala, Stage 2 to TDP-43 pathology in the amygdala and hippocampus, and Stage 3 to TDP-43 pathology in the amygdala, hippocampus, and middle frontal gyrus.

*HS:* HS was unilaterally assessed by H&E staining in the medial temporal lobe structures, and selective neuronal loss and gliosis/sclerosis were assessed. For this study HS scoring was evaluated as present or absent.

Aging-related tau astrogliopathy (ARTAG): ARTAG was evaluated across all available tau slides for each case. ARTAG was scored as present or absent; however, assessment was done in 57/63 cases, and not all brain regions recommended by the guidelines [57] were available for evaluation. These findings should be considered exploratory.

### Clinical evaluation

Individuals enrolled in the ADRC and ABC-DS cohorts underwent several cognitive assessment cycles. During each assessment cycle, dementia was determined at a Consensus Case Conference involving at least three experts with clinical training in dementia diagnosis [36]. During these meetings, the following information was reviewed: medical and psychiatric history, neurological examination, core informant reviews, and the participant's performance on a core battery of direct tests. Instead of using specific cutoff scores, the overall pattern of change in performance was considered. Major life events, psychiatric and medical conditions, and baseline IQ are considered when evaluating change in performance. Following the recommendations of the American Association on Mental Retardation-International Association for the Scientific Study of Intellectual Disability (AAMR-IASSID) Working Group for the Establishment of Criteria for the Diagnosis of Dementia in Individuals with Developmental Disability [6, 18], individuals with DS were classified into four groups: (1) cognitively stable (without dementia), defined as individuals without cognitive or functional decline; (2) Mild cognitive impairment (MCI)-DS, encompasses those individuals that have some cognitive and/or functional decline that is greater than expected with normal aging but that does not meet criteria for dementia; (3) possible dementia was used when the presentation of dementia is aberrant or is concomitant with secondary disorders that may produce dementia and (4) dementia, defined as those individuals with DS with a history of progressive memory loss, functional decline and disorientation over a minimum period of one year after ruling out other non-medical or psychiatric conditions that may confound dementia diagnosis. The clinical diagnosis used in this study was the last diagnosis before death. For this study, we analyzed the frequency of co-pathologies in individuals who were diagnosed as cognitively stable (without dementia) and those with dementia.

### *APOE* genotyping

DNA was extracted from frozen postmortem human brain tissue (occipital lobe or cerebellum) using the QIAamp DNA mini kit (Qiagen) and the Qiagen QIAcube Connect RNA/DNA Extraction Instrument, according to manufacturer’s instructions. The DNA was then quantified and assessed for purity using a Nanodrop spectrophotometer. The RT-PCR was performed using the TaqMan Single SNP Assay kit with TaqMan primers rs7412 (Thermofisher 4,351,379, C___904973_10) and rs429358 (Thermofisher 4,351,379, C___3084793_20). Fluorogenic allele-specific oligonucleotide probes were used for automated genotype detection for all three *APOE* alleles: ε2, ε3, and ε4 as described in [53]. The amplification protocol consists of one ten-minute pre-incubation cycle at 95 °C followed by 40 amplification cycles, each consisting of a ten-second denaturing cycle at 95 °C and a one-minute and 20-s hybridization cycle at 60 °C [53]. The results were analyzed using the LightCycler ® 480 Software version 1.5.

### Karyotyping

Karyotype information was available for all DS cases from medical records or karyotyping at birth or upon enrollment in the ABC-DS study, as described in [37]. Individuals with DS with mosaicism or partial trisomy 21 were not included in this report; thus, all the cases in this study had full trisomy of chromosome 21.

### Premorbid intellectual disability

Premorbid intellectual disability (ID) was assessed before the onset of cognitive impairment. Classification was based on Stanford-Binet Intelligence Scales, Fifth Edition (SB5; Abbreviated Battery) [104], or from medical records documenting IQ with the Wechsler Adult Intelligence Scale [122] or Kaufman Brief Intelligence Test [52]. Premorbid ID was categorized as mild (1), moderate (2), or severe/profound (3), according to IQ standard score ranges (mild: 50–69; moderate: 35–49; severe/profound: < 35) or age-equivalent scores (mild: 9–14 years; moderate: 4–8 years; severe/profound: ≤ 3 years). For individuals diagnosed with AD dementia, we used the IQ or mental age equivalent score obtained before dementia onset.

### Statistics

Data were analyzed with GraphPad Prism 10.2.2. For comparisons between individuals with DS with and without dementia, two-tailed unpaired Student’s *t*-tests or Mann–Whitney tests were used according to data distribution (Shapiro–Wilk normality test). Categorical variables were assessed by performing a two-sided Fisher’s exact test. Missing values for each outcome measure were excluded from the analyses. Significance was set at *P* < 0.05.

## Results

The demographic and clinical characteristics of this study cohort are summarized in Table 1. Figure 1 presents all the neuropathological findings for each individual. A total of 63 adults with DS were included, comprising 25 women (40%) and 38 men (60%), with a mean (SD) age of 56 ± 6.2 years (range: 42 – 70 years). Most individuals had mild (32%) or moderate (42%) premorbid intellectual disability, with 16 (26%), classified as severe/profound. The cohort was predominantly white (89%). Most individuals carried the *APOE* ɛ3/ɛ3 genotype (59%), followed by ɛ3/ɛ4 (28%), ɛ2/ɛ3 (8%), ɛ4/ɛ4 (3%), and ɛ2/ɛ4 (2%).

Following consensus diagnosis, 55 individuals with DS were diagnosed with dementia (mean [SD] age, 56 [5.5] years), and eight did not have dementia (mean [SD] age, 60 [9.4] year; cases #1, 13, 47, 49, 51, 56, 62, and 63). The time between the last clinical evaluation and death was (median [IQR]) 9 [6.4–12.2] months for those without dementia and 8.5 [3.1–22.4] months for those with dementia. Age did not differ significantly between groups (*P* = 0.07). Sex, premorbid intellectual disability, and APOE genotype distribution were all comparable between individuals with and without dementia.

### Gross and microscopic findings in adults with Down syndrome

**Fig. 2:**
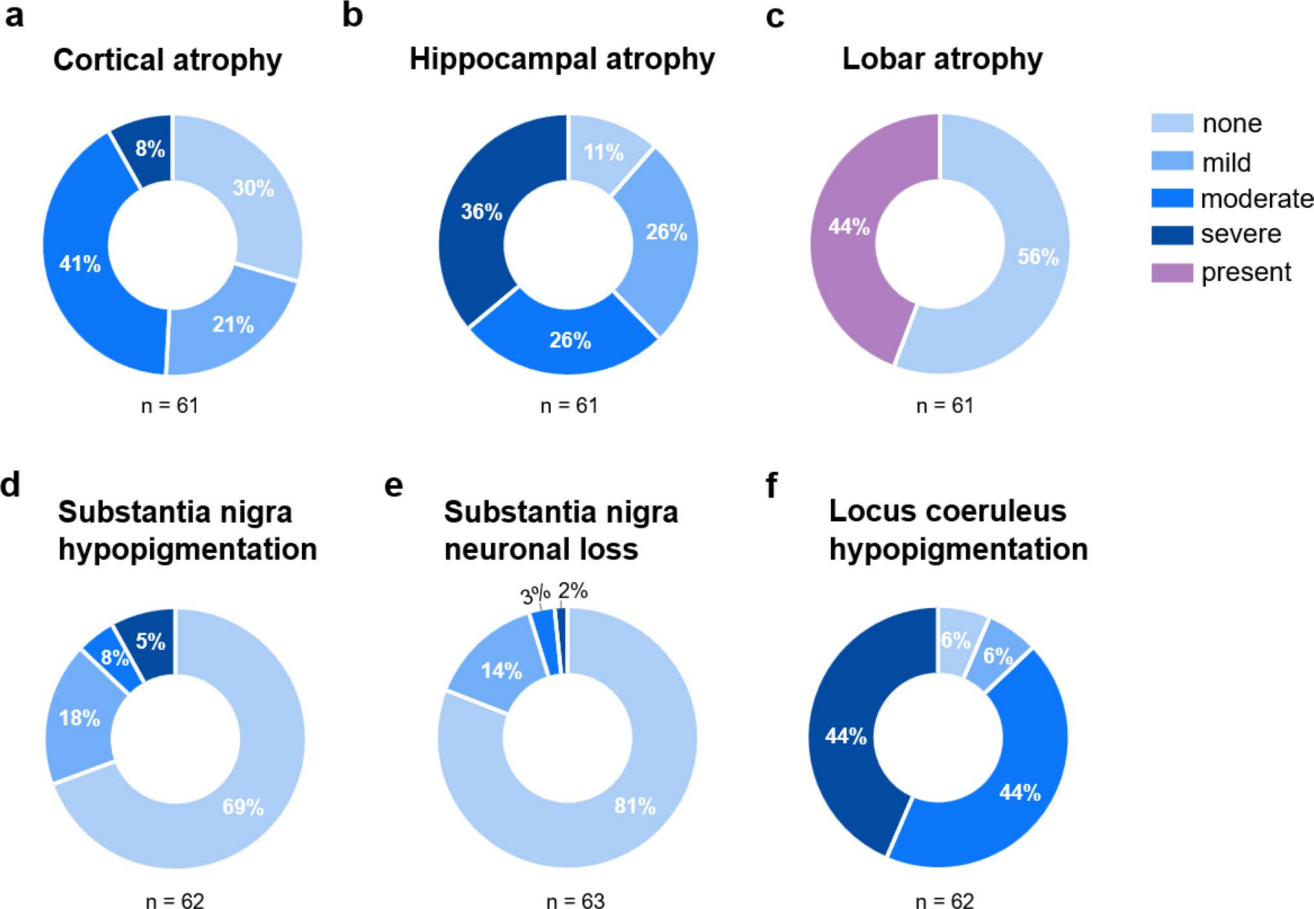
Gross neuropathological findings in individuals with DS. **a–f** Donut charts illustrating the frequency of gross neuropathological findings across the DS autopsy cohort.** a** Cortical atrophy was present in most individuals with DS. **b** Hippocampal atrophy was predominantly moderate to severe. **c** Lobar atrophy was present in 44% of adults with DS, the predominant pattern was superior temporal gyrus atrophy. **d–e** Substantia nigra hypopigmentation and neuronal loss were absent in most individuals. **f** In contrast, moderate to severe locus coeruleus was a frequent finding

Gross findings, including brain weight, cerebral atrophy, substantia nigra and locus coeruleus hypopigmentation, and substantia nigra neuronal loss, are outlined in Table 2 and Fig. 2. Exploratory comparisons between individuals with and without dementia are presented in Supplemental Fig. S1.

**Table 2:** Gross and microscopic findings in individuals with Down syndrome

	All DS n (%)	DS without dementia n (%)	DS with dementia n (%)	*P* value
Fresh brain weight (n)	58	8	50	
Mean (SD), grams	922 ± 127	1060 ± 108	900 ± 116	**0.0006**
Range	685–1217	887–1217	685–1196	
Cortical atrophy (n)	61	8	53	
None	18 (30)	5 (63)	13 (25)	0.13
Mild	13 (21)	2 (25)	11 (21)	
Moderate	25 (41)	1 (13)	24 (45)	
Severe	5 (8)	0	5 (9)	
Lobar atrophy (n)	61	8	53	
Present	27 (44)	2 (25)	25 (47)	0.28
Absent	34 (56)	6 (75)	28 (53)	
Hippocampal atrophy (n)	61	8	53	
None	7 (12)	3 (38)	4 (14)	**0.049**
Mild	16 (26)	3 (38)	13 (46)	
Moderate	16 (26)	1 (13)	15 (54)	
Severe	22 (36)	1 (13)	21 (75)	
Substantia nigra hypopigmentation (n)	62	8	54	
None	43 (69)	6 (75)	37(69)	0.62
Mild	11 (18)	1 (13)	10 (19)	
Moderate	3 (5)	1 (13)	2 (4)	
Severe	5 (8)	0	5 (9)	
Substantia nigra neuron loss (n)	63	8	55	
None	51 (81)	7 (83)	44 (69)	> 0.99
Mild	9 (14)	1 (13)	8 (19)	
Moderate	2 (3)	0	2 (4)	
Severe	1 (2)	0	1 (9)	
Locus coeruleus hypopigmentation (n)	62	8	54	
None	4 (6)	2 (25)	2 (4)	**0.009**
Mild	4 (6)	1 (13)	3 (6)	
Moderate	27 (44)	5 (63)	22 (41)	
Severe	27 (44)	0	27(50)	

The first column presents findings for the full DS autopsy cohort (All DS). The following columns present findings stratified by dementia status and group comparisons. DS = Down syndrome. Some percentages do not total 100 due to rounding

**Fig. 3:**
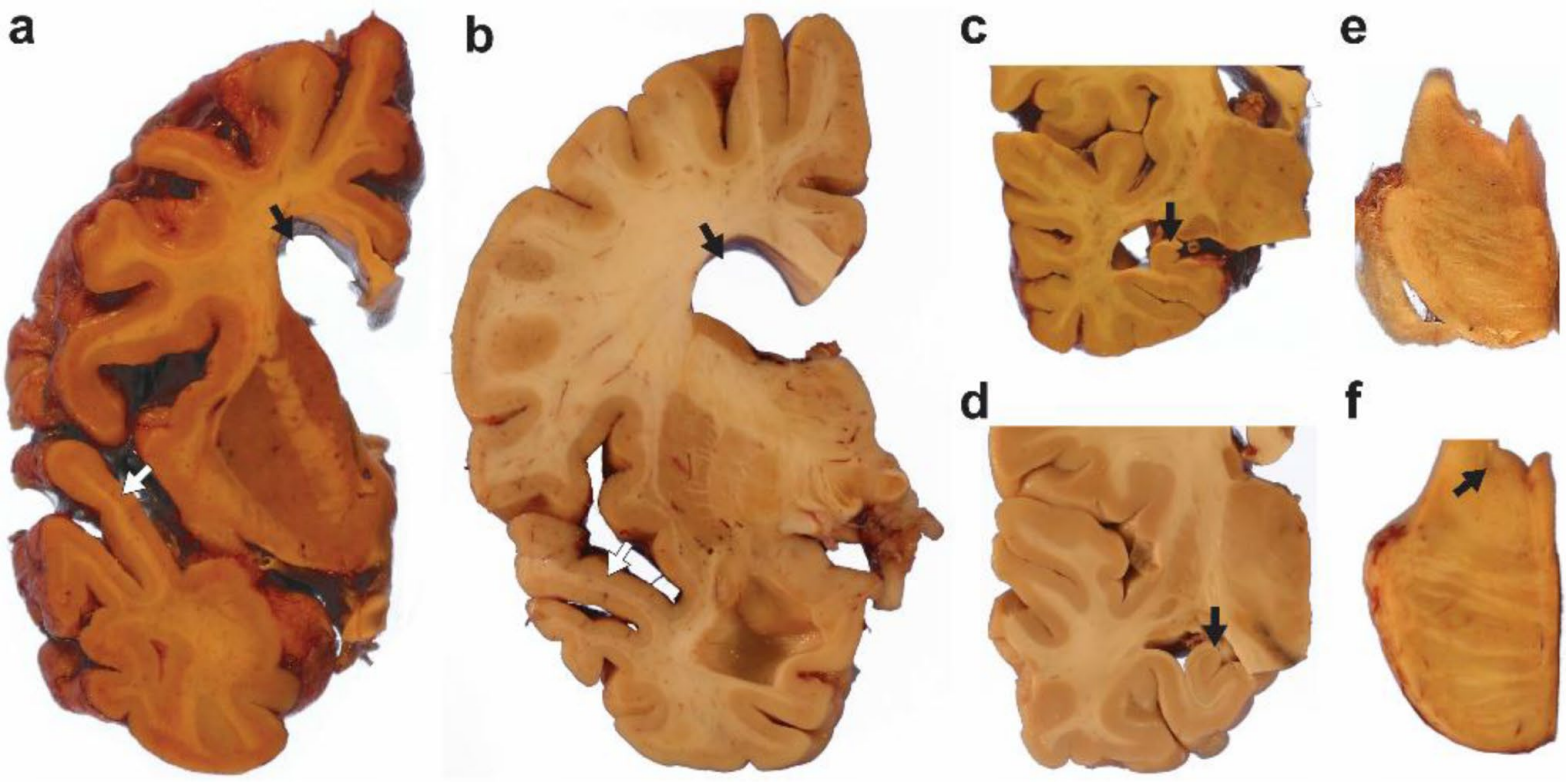
Representative macroscopic neuropathologic findings in individuals with DS with and without dementia. **a** Coronal section demonstrating diffuse moderate cerebral cortical atrophy as evidenced by enlarged sulci and hydrocephalus ex vacuo (black arrow) in addition to atrophy of the superior temporal gyrus (white arrow) to a greater degree than the rest of the temporal lobe. **b** Coronal section highlighting temporal lobe atrophy, especially of the superior temporal gyrus (white arrow), and a moderately dilated lateral ventricle (black arrow). **c–d** Hippocampi from individuals with DS with (c) and without (d) dementia demonstrating the degree of hippocampal atrophy observed in both groups. **e–f** Locus coeruleus hypopigmentation in individuals with DS with (e) and without (f) dementia demonstrating hypopigmentation in both groups but more severe pathology in cases with dementia

The average brain weight for all individuals with DS was 922 ± 127 g and was significantly lower in individuals with dementia (mean [SD] weight, 900 [116] grams) compared to those without (mean [SD] weight, 1060 [108] grams; *P* = 0.0006). Mild, moderate and severe cortical atrophy was present in 70% of the cohort, and lobar atrophy in 44%, with no significant differences between dementia groups. Review of the neuropathological reports revealed that superior temporal gyrus atrophy was predominant in this cohort and was recorded as lobar atrophy in the NACC forms (Fig. 3a-b). Severe hippocampal atrophy was more frequent in individuals with dementia (*P* = 0.049) (Fig. 3c-d). Substantia nigra hypopigmentation and neuronal loss were rare overall and similarly distributed across groups. In contrast, moderate-to-severe LC hypopigmentation was a common finding, affecting 54/63 (88%) of individuals, and severe hypopigmentation was significantly more frequent in those with dementia (*P* = 0.009) (Fig. 3e-f).

### Alzheimer’s disease pathology in adults with Down syndrome

**Fig. 4:**
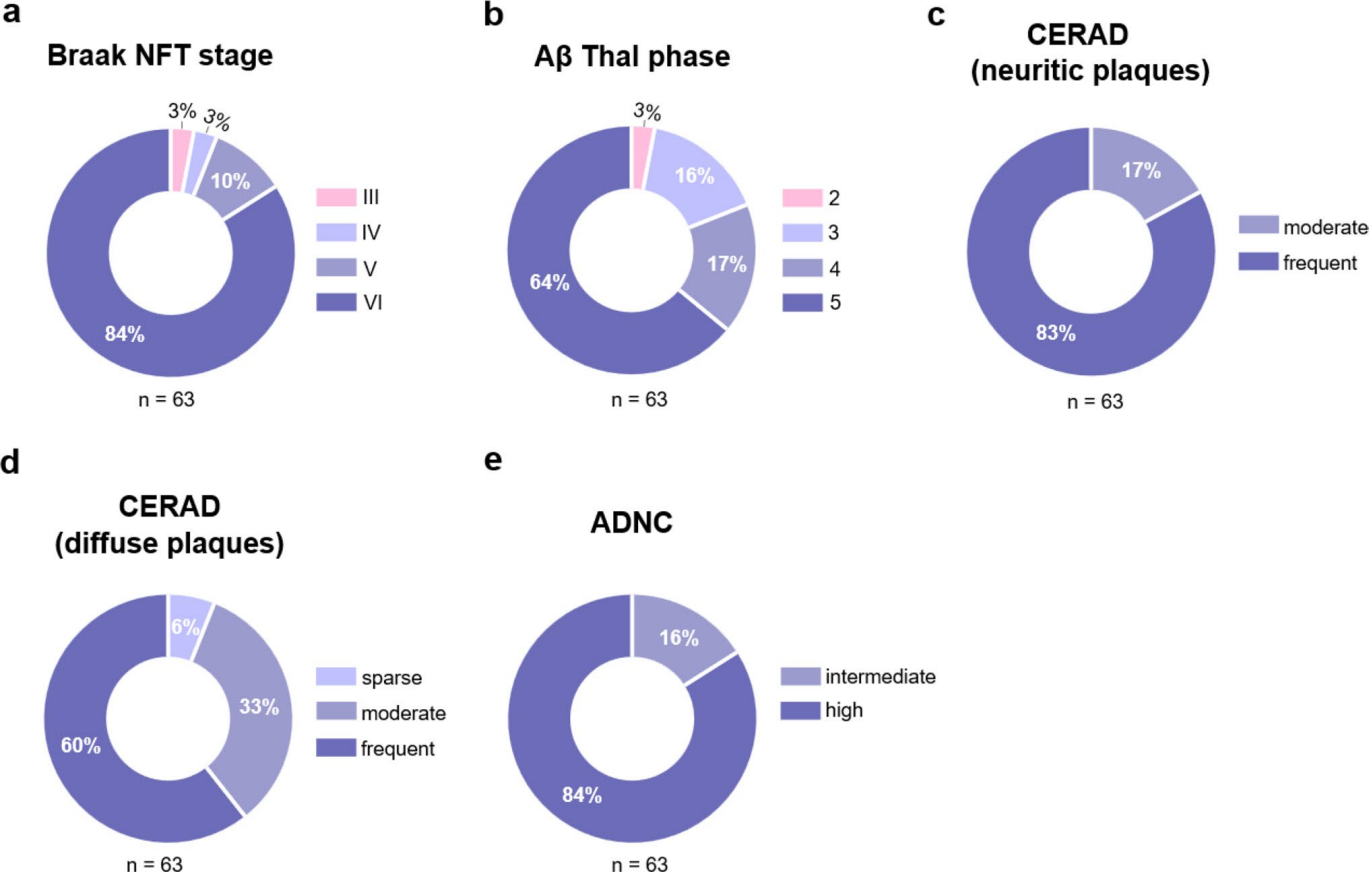
Alzheimer’s disease neuropathological change in individuals with DS. **a–e** Donut charts illustrating the frequency of ADNC across the DS autopsy cohort. **a–b** Most individuals had advanced Braak NFT and Thal phases, and frequent neuritic and diffuse plaques (**c–d**). **e** Most adults with DS over age 40 years had high ADNC. NFT = neurofibrillary tangles, CERAD = Consortium to Establish a Registry for Alzheimer’s disease, ADNC = Alzheimer’s disease neuropathological change

Alzheimer’s disease neuropathological findings for all individuals with DS are presented in Table 3 and Fig. 4. Supplemental Fig. S2 presents findings on ADNC in individuals with DS stratified by dementia status. Most individuals with DS showed advanced Braak NFT stages, with 59 (94%) at Braak stage V-VI and four reaching Braak stages III-IV.

**Table 3:** Alzheimer’s disease neuropathological change in individuals with Down syndrome

	All DS n (%)	S without dementia n (%)	DS with dementia n (%)	P value
n	63	8	55	
Braak NFT stage				
III	2 (3)	2 (25)	0	**0.0001**
IV	2 (3)	2 (25)	0	
V	6 (10)	1 (13)	5 (9)	
VI	53 (84)	3 (38)	50 (91)	
Thal phase				
2	2 (3)	0	2 (4)	0.19
3	10 (16)	2 (25)	8 (15)	
4	11 (17)	3 (38)	8 (15)	
5	40 (64)	3 (38)	37 (67)	
CERAD score (neuritic)				
Moderate neuritic plaques	11 (17)	4 (50)	7 (13)	**0.03**
Frequent neuritic plaques	52 (83)	4 (50)	48 (87)	
CERAD score (diffuse)				
Sparse diffuse plaques	4 (6)	0	4 (7)	> 0.99
Moderate diffuse plaques	21 (33)	3 (38)	18 (33)	
Frequent diffuse plaques	38 (60)	5 (62)	33 (60)	
NIA-AA ADNC				
Intermediate ADNC	10 (16)	5 (63)	5 (9)	**0.0016**
High ADNC	53 (84)	3 (38)	50 (91)	

*DS* Down syndrome, *CERAD* Consortium to Establish a Registry for Alzheimer’s disease, *ADNC* Alzheimer’s disease neuropathological change, *NIA-AA* National Institute on Aging and Alzheimer’s Association

The first column presents findings for the full DS autopsy cohort (All DS). The following columns present findings stratified by dementia status and group comparisons.. Some percentages do not total 100 due to rounding

**Fig. 5:**
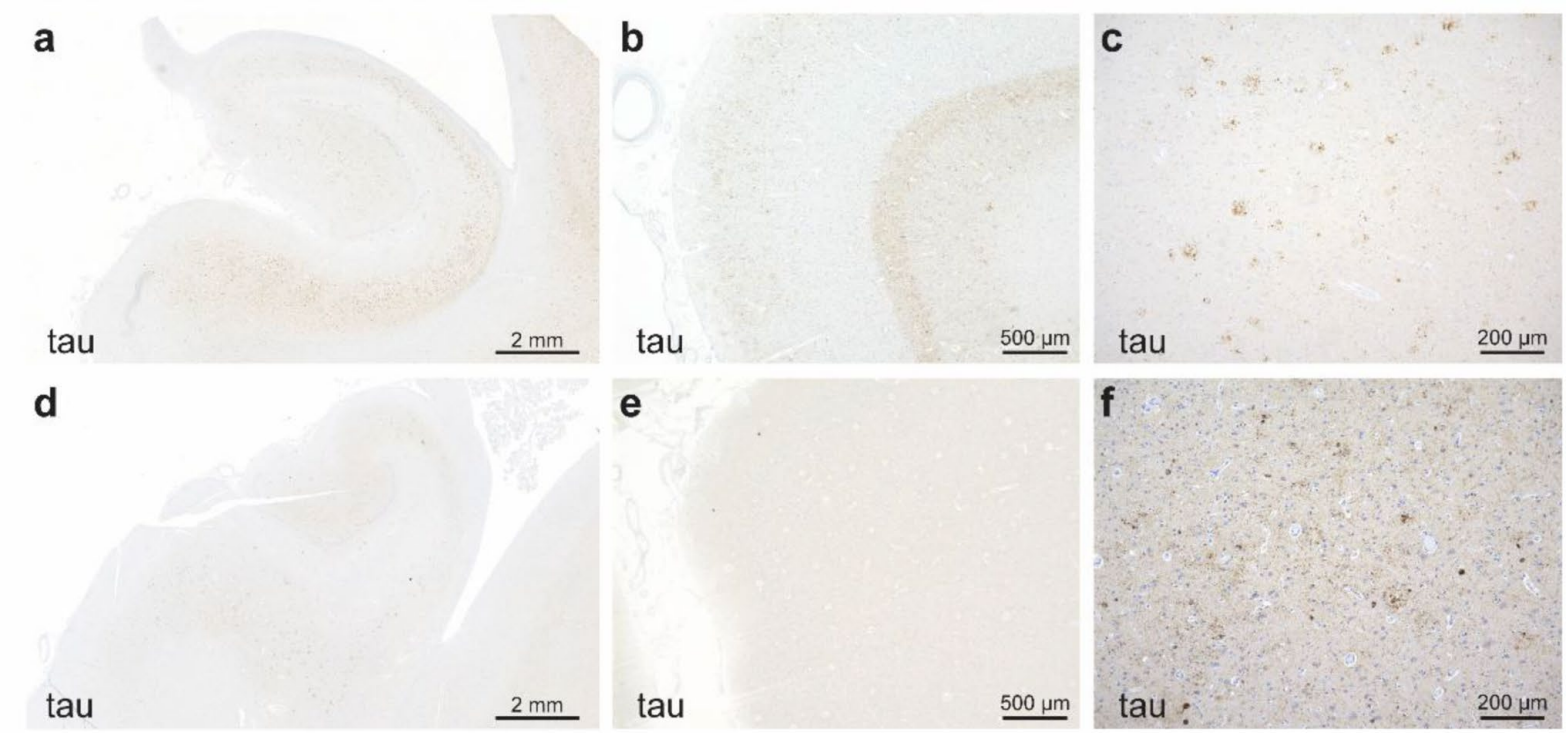
Representative microscopic neuropathologic findings in individuals with Down syndrome with and without dementia. **a—f** Tau immunostained sections from individuals with DS with (a—c) and without (d—f) dementia. Low power images of hippocampi from DS individuals with (a) and without (d) dementia demonstrate the increased burden of tau pathology in individuals with dementia. Images of the striate/primary visual cortex in the occipital lobe from individuals with (b) and without (e) dementia show the high burden and Braak stage (Braak stage VI) that was commonly observed in people with dementia. Images of the middle frontal lobe cortex from individuals with (c) and without (f) dementia demonstrating significant neuritic plaque pathology in both groups but with a greater burden/CERAD score in individuals with dementia

Braak NFT stage distribution differed significantly between dementia groups (*P* = 0.0001), with half of individuals without dementia (cases #13, 47, 49, and 56) presenting with lower Braak stages (III-IV) and the other half (cases #1, 51, 62, and 63) with advanced stages (V-VI), compared to those with dementia, among whom 99% reached stages V-VI (Fig. 5a, b, d, e).

Thal phase distribution did not differ significantly between dementia groups, with most individuals across both groups reaching phase 4 and 5. Similarly, CERAD diffuse plaque scores were comparable between those with and without dementia. In contrast, CERAD neuritic plaque scores differed significantly between dementia groups (*P* = 0.03), with frequent neuritic plaques observed in 87% individuals with dementia compared to 50% of those without (Fig. 5c,f). Consistent with this, NIA-AA ADNC classification differed significantly between dementia groups (*P* = 0.0016), with 91% of those with dementia presenting high ADNC compared to 38% of those without dementia.

### Cerebral amyloid angiopathy is common in adults with Down syndrome

**Fig. 6:**
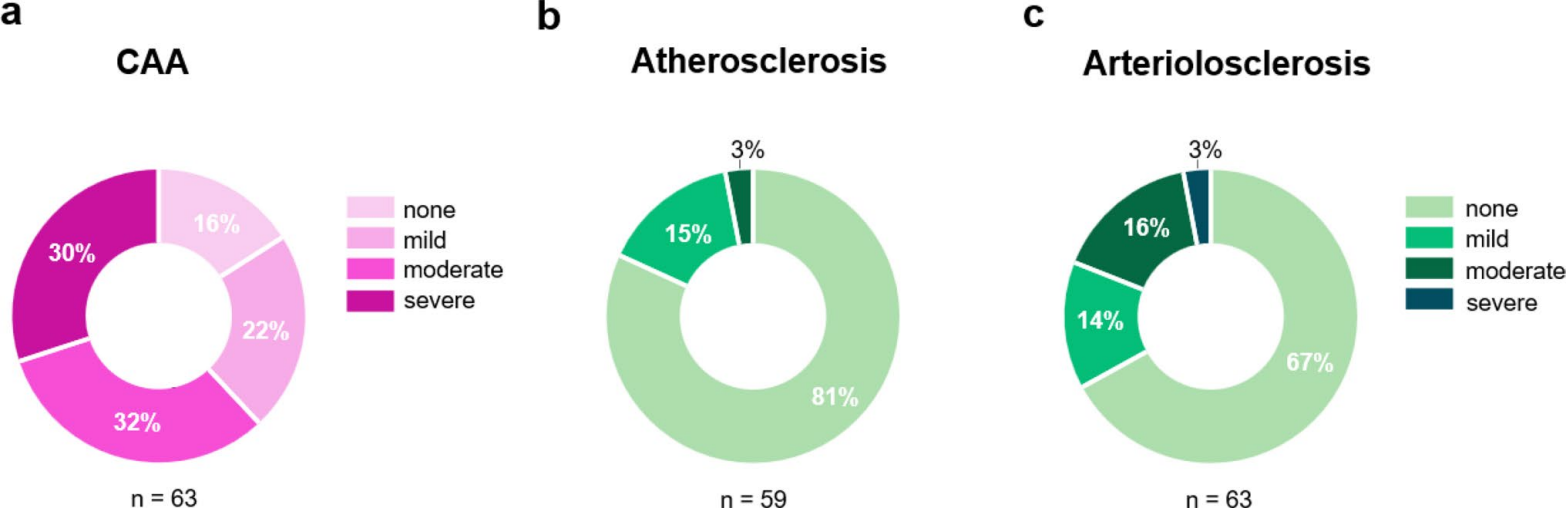
Frequency of CAA, atherosclerosis, and arteriolosclerosis in individuals with DS. **a–c** Donut charts illustrating the frequency of cerebrovascular pathology across the DS autopsy cohort. **a** Most individuals with DS had mild to severe CAA. **b–c** Atherosclerosis and arteriolosclerosis were not frequent findings in adults with DS

CAA findings are outlined in Table 4 and Fig. 6a. CAA was present in most individuals with DS, with 62% presenting moderate or severe forms of CAA and 22% with mild CAA. Only ten individuals (16%) showed no CAA. CAA severity was similarly distributed between individuals with and without dementia (*P* = 0.40) (Supplemental Fig. S3a). Given prior reports of increased CAA severity in APOE ε4 carriers in the non-DS population, we examined whether this association was present in our cohort. *APOE* ε4 carriers did not display a higher frequency of moderate-to-severe CAA compared to non-carriers (*P* > 0.27; Supplemental Table S1).

**Table 4:** Cerebral amyloid angiopathy pathology in adults with Down syndrome

	All DS n (%)	DS without dementia n (%)	DS with dementia n (%)	*P* value
CAA	63	8	55	
None	10 (16)	3 (38)	7 (13)	0.40
Mild	14 (22)	1 (13)	13 (24)	
Moderate	20 (32)	2 (25)	18 (33)	
Severe	19 (30)	2 (25)	17 (31)	

*DS* Down syndrome, *CAA* cerebral amyloid angiopathy

The first column presents findings for the full DS autopsy cohort (All DS). The following columns present findings stratified by dementia status and group comparisons. Some percentages do not total 100 due to rounding

### Frequency of other cerebrovascular pathologies in Down syndrome

Vascular pathology findings are summarized in Table 5 and Fig. 6b-c. Overall, most cerebrovascular findings were absent or mild across all pathology types. Mild atherosclerosis changes were present in 9 (15%), moderate in 2 (3%) and absent in 48 (81%) individuals. All individuals without dementia had no atherosclerosis, compared to 41 (79%) of those with dementia; however, this difference was not statistically significant (*P* = 0.68) (Supplemental Fig. S3b). Arteriolosclerosis was also infrequent, with mild, moderate, and severe changes present in 9 (14%), 10 (16%), and 2 (3%) individuals, respectively and absent in 42 (67%). Arteriolosclerosis severity distribution was comparable between dementia groups (Supplemental Fig. S3c). Similarly, infarcts, microinfarcts, and hemorrhages were not common findings in this autopsy cohort. Infarcts were present in 4 individuals (6%). Infarcts were gross/large in all cases. Two chronic or old infarcts were identified in individuals with dementia, affecting the periventricular white matter (case #2) and the gray and white matter of the mid frontal gyrus (case #24), respectively. The remaining two occurred in individuals without dementia, one large subacute infarct involving gray and white matter in the territory of the middle cerebral artery (case #51) and one chronic or old infarct in the cerebellar gray matter (case #63). Three unilateral subacute microinfarcts in the frontal and occipital lobes were identified in a single individual with dementia, two in the gray matter and one in the white matter (case #54). White matter rarefaction was present in 16 (24%) of all individuals, with a similar distribution in those with and without dementia. Vessel mineralization data were available in a subset of cases (four without dementia and 14 with dementia) and are reported as preliminary findings in Supplemental Table S2. Among those with available data, vessel mineralization was present in half of the individuals without dementia (2/4) and 13/14 of those with dementia.

**Table 5:** Cerebrovascular pathology in adults with Down syndrome

	All DS n (%)	DS without dementia n (%)	DS with dementia n (%)	P value
Atherosclerosis	59	7	52	
None	48 (81)	7 (100)	41 (79)	0.68
Mild	9 (15)	0	9 (17)	
Moderate	2 (3)	0	2 (4)	
Arteriolosclerosis	63	8	55	
None	42 (67)	5 (63)	37 (67)	0.79
Mild	9 (14)	2 (25)	7 (13)	
Moderate	10 (16)	1 (13)	9 (16)	
Severe	2 (3)	0	2 (4)	
Infarcts	63	8	55	
Present	4 (6)	2 (25)	2 (4)	0.08
Absent	59 (9)	6 (75)	53 (96)	
Microinfarcts	63	8	55	
Present	1 (2)	0	1 (2)	> 0.99
Absent	62 (98)	8 (100)	54 (98)	
Hemorrhages	62	8	54	
Present	4 (6)	2 (25)	2 (4)	0.08
Absent	58 (94)	6 (75)	52 (96)	
White matter rarefaction	63	8	55	
Present	16 (24)	2 (25)	14 (25)	> 0.99
Absent	47 (75)	6 (75)	41 (75)	
Vessel mineralization	18	4	14	
Present	15 (83)	2 (25)	13 (93)	0.11
Absent	3 (17)	2 (50)	1 (7)	

*DS* Down syndrome

The first column presents findings for the full DS autopsy cohort (All DS). The following columns present findings stratified by dementia status and group comparisons. Some percentages do not total 100 due to rounding

### Frequency of other neuropathologies in Down syndrome

**Fig. 7:**
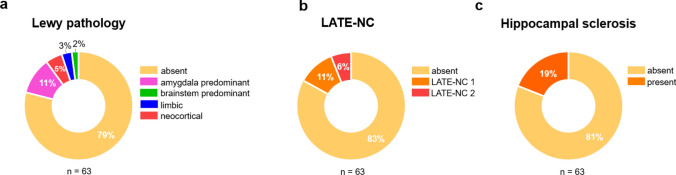
Frequency of Lewy pathology, LATE-NC, and hippocampal sclerosis in individuals with DS. **a–c** Donut charts illustrating the frequency of neuropathologies across the DS autopsy cohort. **a** Lewy pathology was present in 21% of the autopsy cohort, and was mostly identified in the amygdala.** b** LATE-NC was identified in 17% of individuals with DS, with the majority at stage 1. **c** Hippocampal sclerosis was present in 19% of individuals. LATE-NC = limbic predominant age-related TDP-43 encephalopathy neuropathological change

Co-pathology findings are summarized in Table 6 and Fig. 7. Overall, LP, LATE-NC (TDP-43 pathology), and HS were present in a minority of adults with DS, and none differed significantly between dementia groups (Supplemental Fig. S4).

**Table 6:** Lewy pathology, LATE-NC, and hippocampal sclerosis in individuals with Down syndrome

	All DS n (%)	DS without dementia n (%)	DS with dementia n (%)	*P* value
n	63	8	55	
Lewy pathology				
Present	13 (21)	2 (25)	11 (20)	0.66
Absent	50 (79)	6 (75)	44 (80)	
Category				
Amygdala predominant	7 (11)	2 (25)	5 (9)	
Brainstem predominant	1 (2)	0	1 (2)	
Limbic	2 (3)	0	2 (4)	
Neocortical	3 (5)	0	3 (5)	
TDP-43				
Present	11 (17)	0	11 (20)	0.33
Absent	52 (83)	8 (100)	44 (80)	
LATE-NC stage				
1	7 (11)	0	7 (13)	
2	4 (6)	0	4 (7)	
Hippocampal sclerosis				
Present	12 (19)	0	12 (22)	0.33
Absent	51 (81)	8 (100)	43 (78)	

*DS* Down syndrome, *TDP-43* transactivase response DNA-binding protein 43 kDa, *LATE-NC* Limbic-predominant age-related TDP-43 encephalopathy neuropathological change

The first column presents findings for the full DS autopsy cohort (All DS). The following columns present findings stratified by dementia status and group comparisons

LP was present in 13 (21%) adults with DS, with comparable frequencies in those without (25%) and with dementia (20%, P = 0.66). When present, the most frequent subtype in both groups was amygdala predominant, identified in 7 (11%) of all cases, followed by neocortical in 3 (5%), limbic in 2 (3%), and brainstem predominant in 1 (2%). All LP subtypes occurred in individuals with dementia, while individuals without dementia had amygdala predominant only pathology.

TDP-43 pathology was present in 11 (17%) individuals with DS, of whom all had dementia. Among those with TDP-43 pathology, LATE-NC stage 1 and stage 2 were observed in seven and four individuals, respectively. Hippocampal sclerosis was identified in 12 (19%) individuals and was only present in those with dementia. TDP-43 pathology was absent in most cases with HS. Three individuals had concurrent TDP-43 pathology, two were classified as LATE-NC stage 1 (cases #35, and 42) and one as LATE-NC stage 2 (case #61). Although LATE-NC and HS were identified only in those with dementia, this difference did not reach statistical significance.

ARTAG was assessed in a subset of cases (*n* = 57) based on available tau immunostaining slides; regional sampling was not uniform across cases due to the relatively new addition of this assessment to the NACC forms, the retrospective nature of our cohort and differences in available tau-stained slides. Therefore, these findings are only exploratory. In this cohort, ARTAG was an uncommon finding, present in 4 (7%) individuals, all of whom had dementia (Supplemental Table S3). No significant differences were found between dementia groups.

### Concomitant pathologies in the brains of adults with Down syndrome

**Fig. 8:**
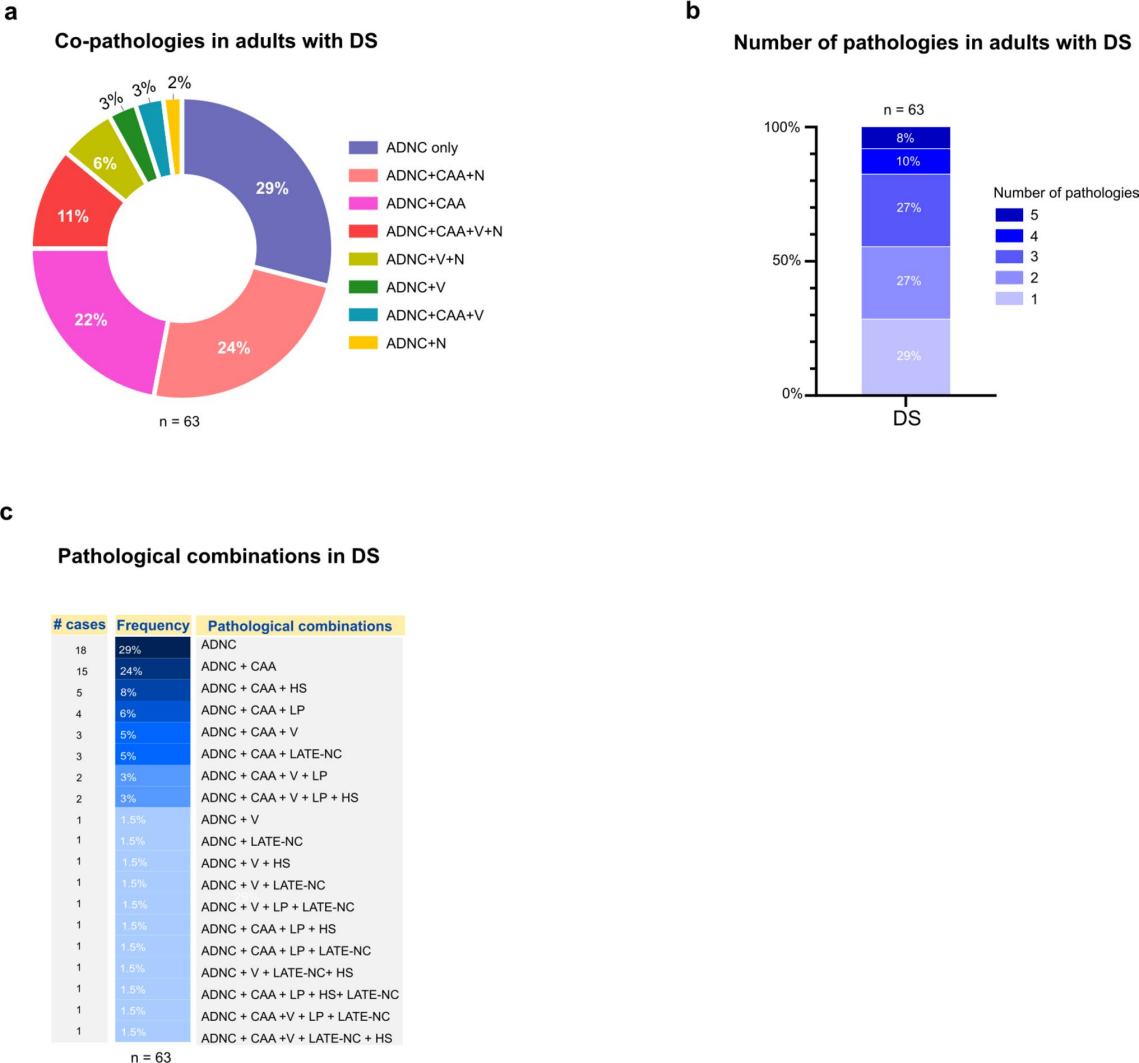
Mixed neuropathologies in adults with Down syndrome. **a** Donut chart illustrating the distribution of co-pathologies in the full DS autopsy cohort. Cases were classified into eight groups based on the presence of co-pathologies: ADNC only, CAA (moderate or severe), vascular pathology (V; moderate or severe atherosclerosis or ateriolosclerosis), and other neuropathologies (N; Lewy pathology, LATE-NC, and/or hippocampal sclerosis). **b** Stacked bar chart illustrating the distribution of the total number of individual co-pathologies (ADNC, CAA, V, atherosclerosis/arteriosclerosis, Lewy body pathology, LATE-NC, hippocampal sclerosis). Most individuals carried one to three pathologies.** c** Table illustrating the pathological combinations identified in the cohort. The most common combinations were ADNC only, ADNC + CAA, and ADNC + CAA + HS. ADNC = Alzheimer’s disease neuropathological change, CAA = cerebral amyloid angiopathy, LP = Lewy pathology, HS = hippocampal sclerosis, LATE-NC = limbic predominant age-related TDP-43 encephalopathy neuropathological change

To characterize co-pathologies in this autopsy cohort, we classified cases according to the presence or absence of the next pathological categories concurrent with ADNC: (1) CAA (moderate or severe), (2) vascular pathology (V; moderate or severe atherosclerosis or arteriolosclerosis), and (3), neurodegenerative co-pathologies (N; presence of LP, and/or LATE-NC, and/or HS). Each case was assigned to one of eight groups based on all present combinations, ranging from ADNC only to ADNC with all three co-pathologies (ADNC + CAA + V + N). The frequency of each combination across the full cohort and by dementia status is summarized in Table 7 and Fig. 8a.

**Table 7:** Frequency of co-pathologies in adults with Down syndrome

	All DS n (%)	DS without dementia n (%)	DS with dementia n (%)	*P* value
n	63	8	55	
ADNC only	18 (29)	4 (50)	14 (25)	0.88
ADNC + CAA	14 (22)	2 (25)	12 (22)	
ADNC + V	2 (3)	0	2 (4)	
ADNC + CAA + V	2 (3)	0	2 (4)	
ADNC + N	1 (2)	0	1 (2)	
ADNC + CAA + N	15 (24)	1 (13)	14 (25)	
ADNC + V + N	4 (6)	0	4 (7)	
ADNC + CAA + V + N	7 (11)	1 (13)	6 (11)	

*DS* Down syndrome, *ADNC* Alzheimer’s disease neuropathologic change, *CAA* moderate or severe cerebral amyloid angiopathy, *V* moderate and/or severe atherosclerosis or arteriolosclerosis, *N* Lewy body pathology and/or TDP-43 pathology and/or hippocampal sclerosis

Some percentages do not total 100 due to rounding

Pure ADNC (ADNC only), in the absence of any additional pathology, was present in a minority of the cohort in only 18 (29%) individuals. Most individuals with DS had at least one additional co-pathology co-occurring with ADNC. CAA (moderate-to-severe) was the most frequent co-pathology, present in 38 out of 63 cases (60%) either alone or in combination with other pathologies. The most frequent neuropathological combination was ADNC + CAA + other neuropathology (LP, LATE-NC or HS), observed in 15 (24%) individuals, followed by ADNC + CAA in 14 (22%) individuals. Vascular pathology (moderate-to-severe atherosclerosis or arteriolosclerosis) rarely occurred as an isolated co-pathology, being present in 2 (3%) individuals (ADNC + V).

Although not significant, when comparing groups by dementia status, pure ADNC was observed in half of individuals without dementia compared to only 25% of those with dementia (Supplemental Figure S5a). In addition, CAA was identified as the main co-pathology in all individuals without dementia. The distribution of co-pathologies in those with dementia was similar to what was found in the overall DS group.

Finally, the categorical approach that we used grouped LP, LATE-NC, and HS into a single group (N). While this approach has been used in other studies, it does not capture the true number of independent co-pathologies present in each individual. To address this, we counted each pathology separately, including ADNC, CAA (moderate-to-severe), vascular pathology (moderate-to-severe atherosclerosis and arteriolosclerosis), LP, LATE-NC, and HS. Our exploratory analysis revealed that overall, 54% of individuals with DS had two or three pathologies and 18% had four or five pathologies (Fig. 8b). When stratified by dementia status, we identified that individuals without dementia commonly had one or two pathologies (6/8, 75%), with only two individuals having three or four pathologies, respectively. In contrast, half of the dementia group displayed one or two pathologies and the rest ranged from three to five (Supplemental Fig. S5b). We identified 19 pathological combinations in all our autopsy cohort, with ADNC (29%), ADNC + CAA (24%), ADNC + CAA + HS (8%), and ADNC + CAA + LP (6%) being the more common combinations in the full cohort (Fig. 8c). We also observed that in terms of pathological combinations, the group without dementia was more homogenous, compared to the increased number of pathological combinations identified in individuals with dementia (Supplemental Fig. S5c).

## Discussion

The present study provides a comprehensive characterization of the frequency and spectrum of co-pathologies in a large clinically and neuropathologically characterized autopsy cohort of adults with DS over the age of 40 years. It also offers an exploratory comparison of neuropathological profiles between adults with DS with and without dementia. This study has three major findings. First, co-pathologies were identified in 71% of adults with DS, while “pure” AD is found only in 29%, regardless of dementia status. Second, moderate-to-severe CAA is the most common co-pathology in adults with DS, present in 62% of our autopsy cohort. Third, 13% of our cases of adults with DS with full trisomy 21 displaying ADNC and co-pathologies did not have dementia at ages when most individuals with DS develop dementia, with 75% of these individuals being over the age of 60 years at death. These individuals had larger brains, fewer showed severe hippocampal atrophy and LC hypopigmentation, and most had intermediate ADNC. We hypothesize that these individuals may exhibit resilience and resistance mechanisms to cognitive decline.

### Gross findings

Our findings showed that most adults with DS and ADNC have cortical atrophy, which is consistent with previous reports showing cortical atrophy due to AD in DS [17, 88]. The neuropathological reports reviewed in this study, consistently described atrophy of the superior temporal gyrus, a finding that may partly reflect neurodevelopmental differences in DS rather than a purely neurodegenerative process, as neuroimaging studies have reported a smaller superior temporal gyrus white matter volume in young adults with DS in the absence of AD pathology [92]. Mild-to-severe hippocampal atrophy was a common finding in our autopsy cohort. Individuals with DS have smaller hippocampi than those without DS due to neurodevelopmental differences [78] and hippocampal atrophy related to aging and AD has also been well documented in DS [108]. Although gross neuropathological observations are unable to fully disentangle whether reduced hippocampal size reflects neurodevelopmental or neurodegenerative mechanisms, the age of our cohort and the near-universal presence of intermediate to high ADNC make it highly likely that the observed hippocampal atrophy/reduced hippocampal size is linked to AD-related neurodegeneration. Notably, individuals without dementia had larger brain weights and less severe hippocampal atrophy compared to those with dementia. Larger hippocampal volumes in DS are associated with better cognitive performance in the modified cued recall test (mCRT) [108], and it has been hypothesized that larger brain size is associated with greater brain reserve, which may confer higher tolerance for neuropathological damage before clinical symptoms emerge [26, 124]. Therefore, individuals with DS without dementia may have greater brain reserve, enabling them to better withstand the neuropathologic burden and cognitive decline associated with ADNC.

The LC, the main source of noradrenaline in the brain, plays an important role in arousal, memory and learning [73, 109], is among the earliest sites of tau accumulation in AD and DS, and exhibits significant shrinkage and neuronal loss as the disease progresses, which is associated with cognitive decline [16, 23, 43, 47, 118]. LC neuronal loss also occurs in DS [32, 71]. In our autopsy cohort, moderate-to-severe LC hypopigmentation was a frequent finding along with lower frequency of severe LC hypopigmentation in those without dementia. The less severe LC hypopigmentation in individuals without dementia suggests a better-preserved noradrenergic network allowing for engaging some LC-mediated cognitive resilience mechanisms [55, 89]. Beyond its role as a neuromodulator, noradrenaline can also act as an anti-inflammatory and neuroprotective molecule [72]. Individuals with DS exhibit reduced levels of neurotrophic factors and a differential neuroinflammatory process across the AD continuum [31, 45]; whether the LC contributes to cognitive resilience through anti-inflammatory and neuroprotective mechanisms remains to be investigated.

Substantia nigra hypopigmentation and neuronal loss were uncommon findings in our cohort. In our study we found moderate-to-severe SN neuronal loss in only three cases and SN hypopigmentation in eight, aligning with a previous study showing only mild SN neuronal loss in 8/13 DS cases [33]. Although few studies have reported Parkinsonian features in DS (20% of adults with DS in Lai et al.) and the presence of α-synuclein pathology in the SN [13, 58, 90, 120], our findings suggest that nigral degeneration is not a prominent finding in adults with DS.

### ADNC

Our studies are consistent with previous DS autopsy cohorts reporting overt ADNC in adults with DS over 40 years old [25]. As expected, most individuals had advanced Braak NFT stages (94%) and Thal phases (81%), and frequent neuritic and diffuse amyloid plaques. However, when comparing individuals with and without dementia we found differences in Braak NFT staging distribution, neuritic plaque frequency, and overall ADNC severity, with tau pathology emerging as the common pathological thread linking these differences. Clinicopathological studies in the non-DS population show that, in contrast to diffuse plaques, advanced Braak NFT stages and neuritic plaques are often associated with low cognitive performance [38, 69, 82, 86]. Thus, it is not surprising that individuals with DS without dementia had lower frequencies of tau pathology (Braak NFT, CERAD, ADNC/NIA-AA). Among individuals without dementia, two distinct subgroups were identified based on Braak NFT staging: The first subgroup consisted of four individuals with advanced Braak NFT stages (V-VI; cases #1, 51, 62, and 63), displaying a tau burden comparable to that of individuals with dementia. These cases showed high ADNC (with one exception with intermediate ADNC: Thal phase 3, CERAD 2), Thal phase 5, and CERAD 3, along with variable hippocampal atrophy and mild-to-moderate LC hypopigmentation. We hypothesize that these individuals exhibited resilience to cognitive decline: maintaining better-than-expected cognition relative to their age and degree of tau pathology. While it remains challenging to draw definitive conclusions as to why these individuals did not develop dementia despite such significant pathological burden, we hypothesize that each case may reflect a convergence of resilience factors potentially including protective genetic variants, fewer co-occurring medical conditions, better education, and lifestyle factors previously shown to impact cognition in people with DS [30, 111]. Further characterization of these individuals is needed to fully understand their resilience profile. The second subgroup comprised four individuals at intermediate Braak NFT stages (III-IV), displaying lower tau pathology than expected for their age. Two individuals in their early 50s and 60s (cases # 13, and 49) were at Braak NFT stage III (Thal Phase 4, CERAD 3 and 2, respectively), and two in their early 60s (cases #47, and 56) were at Braak NFT stage IV (Thal Phase 3 and 4, CERAD 2); one of these cases has been further characterized in a dedicated case report, which provides a detailed description of their neuropathological and clinical profile [63]. Compared to the advanced Braak stage subgroup, these individuals showed none-to-mild hippocampal atrophy, none-to-moderate LC hypopigmentation, a CERAD score of 2, and mostly intermediate ADNC. Given that cognitive performance is typically associated with advanced Braak NFT stages (V-VI) and frequent neuritic plaques [38, 69, 82, 86], and that biomarker studies in DS support a central role for tau in mediating cognitive decline [40, 112], the intermediate tau pathology observed in this subgroup may have been insufficient to trigger dementia onset. We hypothesize that resistance to neocortical tau accumulation, despite the presence of significant amyloid pathology, contributed to delaying or halting dementia in these individuals. While the mechanisms underlying resistance to neocortical tau accumulation in DS remain unknown, genetic variants limiting tau spreading are likely to play a key role as illustrated by two remarkable cases from the Colombian a presenlin-1 (*PSEN1*) E280A kindred: a woman with the protective Christchurch *APOE3* variant and a man carrying a rare RELN-COLBOS variant, both of whom remained cognitively intact for decades beyond the expected age of dementia onset despite high amyloid burden but with limited tau pathology [3, 67]. Identifying analogous protective genetic variants in individuals with DS warrants further investigation.

### Cerebrovascular pathology

In the general population, ADNC and cerebrovascular disease often coexist with the latter reducing the threshold for dementia [4]. The cardiovascular profile of individuals with DS differs from that of the general population with congenital heart disease affecting approximately 50% of individuals with DS, higher rates of obstructive sleep apnea (OSA), obesity, and sedentary lifestyle all of which are associated with cardiovascular disease which increases the risk for dementia [21, 27, 49]. Notably, individuals with DS have a low prevalence of systemic vascular risk factors including hypertension and atherosclerotic cardiovascular disease [29, 80, 97, 103, 105, 106, 128]. This “protective” vascular profile is probably linked to the low blood pressure observed in this population and may be reflected in the low frequency of atherosclerosis and arteriolosclerosis observed in our cohort and others.

In a previous study of 32 DS brains from our study sample (mean age and range: 55.23 ± 6.6, 42—70 years), 25% had atherosclerosis, and none had arteriolosclerosis [41]. In the current expanded sample, we found that 18% had primarily mild atherosclerosis and 33% of DS brains had arteriolosclerosis, mainly in mild-to-moderate forms. Although both samples are similar in age, the increased frequency of arteriolosclerosis may be due to rater variability. In contrast to atherosclerosis and arteriolosclerosis, CAA is present in most DS brains over 40 years, suggesting that cerebrovascular pathology in DS is predominantly driven by CAA rather than by atherosclerotic or arteriolosclerotic disease.

Consistent with smaller DS autopsy studies, mild, moderate, and severe CAA was present in 84% of our cohort and absent in 16% of adults over age 40 years [19, 25, 41, 44, 70]. Previous studies from our laboratory show that CAA severity in DS is greater than in the population without DS and in LOAD, possibly due to chronic amyloid-beta overexpression and impaired vascular and cellular clearance mechanisms [41]. The presence of an *APOE* ε4 allele is associated with increased CAA severity in AD and the general population [74, 96, 99]. However, in our cohort we did not observe an increased frequency of severe CAA in those carrying the *APOE* ε4 allele. This is in line with neuroimaging studies showing a lack of association between CAA and *APOE* genotype in DS [20]*.* However, the number of microbleeds, a possible consequence of CAA, is increased in individuals with DS carrying the *APOE* ε4 genotype [129]. Given that people with DS have an overproduction of Aβ, this may override the influence of *APOE* ε4 on vascular amyloid accumulation, and thus the effect of this risk factor may be below detectable levels in this population.

Neuroimaging studies show increased vascular injury markers in DS, including evidence of small vessel disease and white matter hyperintensities (WMH) [20, 41, 42, 59, 60, 77, 129]. White matter rarefaction was detected in 25% of our autopsy cohort, which may be associated with aging but also with AD, as in the non-DS population and may be reflected as increased WMH in MRI DS studies [9, 59, 60, 77, 129]. Contrary to what has been reported in neuroimaging and neuropathology studies [42, 59, 60, 77, 129], we observed a low frequency of infarcts, microinfarcts, and hemorrhages in DS. Diagnostic neuropathological evaluation does not involve exhaustive brain sampling, which could lead to an underestimation of the total pathological burden. Finally, while vessel mineralization data was only available for a subset of cases, the high frequency of this pathology observed by us and others may be another contributing factor to cerebrovascular disease and unique to this population [116, 127]. A comprehensive and systematic assessment of vessel mineralization is currently underway in our laboratory and will be reported in the future.

When comparing the small subset of individuals without dementia versus those with dementia, we did not find any differences in the frequency or severity of atherosclerosis and arteriolosclerosis. While atherosclerosis was absent in all individuals without dementia, our sample size limits the statistical power to detect possible differences. Equally important is the fact that atherosclerosis in our cohort was predominantly absent or mild, suggesting that it may not be an important contributor to cognitive decline in this population. Likewise, we did not observe a difference in CAA severity between groups, probably because of the AD genetic contribution present in both groups. However, the semiquantitative nature of our data may have limited our ability to detect subtle differences on CAA burden between groups; quantitative pathology is needed to fully characterize the contribution of CAA to cognitive decline in DS.

In this study we also identified a particularly informative group, individuals with DS who, regardless of cognitive status, do not develop or have very mild forms of CAA. The absence of vascular amyloid in a population that has a genetic driver for amyloid overproduction underscores that overproduction is not sufficient to promote vascular deposition and that in turn, there are other mechanisms possibly affecting its deposition in the vasculature. Such mechanisms may involve differences in amyloid clearance mechanisms, blood–brain barrier breakdown, Aβ 40:42 ratios, among others. Therefore, characterizing the neuropathological profile along with imaging and biofluid biomarkers of this subset of individuals may aid in the development of therapeutic strategies aimed at reducing CAA in people with DS and extended to the broader AD population.

The high frequency of cerebrovascular disease including CAA in DS has important implications for the risk of Amyloid-Related Imaging Abnormalities (ARIA), which are observed in some individuals undergoing amyloid-lowering immunotherapies [107]. Postmortem studies show that an anti-amyloid antibody currently used in amyloid-lowering immunotherapies preferentially binds to vascular amyloid in brains from adults with DS over 43 years of age [48, 66]. Given that individuals with DS have significant cerebrovascular disease, including CAA, emerging by their 40s [41] or earlier [59], they may be at increased risk for vascular complications during Aβ immunotherapy. As clinical trials begin to enroll participants with DS [8, 94], understanding their cerebrovascular profile will be critical for determining the optimal age range and treatment window to maximize benefits while minimizing risks.

### Other neurodegenerative co-pathologies

*LP:* LP has been identified in 6% to 39% of the aging population, 50% to 60% of severe AD, and 27% to 63% of ADAD cases [56, 95]. In our cohort, 21% had LP, consistent with frequencies reported in smaller DS autopsy studies ranging from 17 to 47% [19, 25, 44]. The variability across cohorts may be explained by differences in sample size, clinical versus research cohorts, LP scoring criteria, and number of regions sampled. We did not routinely examine LP in the olfactory bulb, which showed a high frequency of LP in the study where 47% of LP positivity was reported in DS. However, consistent with other ADAD and DS reports [61, 119], LP was predominantly found in the amygdala. The relationship between ADNC and LP is complex and its co-occurrence has been associated with worsening of cognitive decline (for a review please see [7]). It has been suggested that tau pathology is closely associated with LP in the amygdala; which is particularly relevant given the high tau burden observed in DS [81, 114]. Alpha-synuclein seed amplification assays are a promising tool to detect LP; however, their sensitivity varies according to the pathological distribution of LP. As such, a recent study using α-synuclein seeding amplification assays in DS reported 9.2% assay positivity in 270 adults with DS, but failed to detect those with amygdala-predominant LP, suggesting that current assays are promising but are not yet sensitive to detect LP in the amygdala [12].

*LATE-NC and HS:* In our study we identified LATE-NC in 17% of adults with DS and it was most predominant in the amygdala, consistent with previous studies reporting this pathology in 10% to 20% DS cases [19, 25, 44, 65, 123] and in 9 to 14% of ADAD and 8% of early-onset AD (EOAD) brains [24, 65]. In the general and sporadic AD populations, LATE-NC is found in higher proportions in ~ 30% of people over 85 years [83] and ~ 50% of end-stage AD cases [85]. The lower frequency of LATE-NC in DS, ADAD, and EOAD compared to sporadic AD or the aging population, may reflect the younger ages of these populations. Indeed, in our cohort, LATE-NC occurred only in individuals over 50 years of age with high ADNC, suggesting that LATE-NC may be primarily age-related rather than an AD neuropathology-driven process in DS. Similar to LATE-NC, HS was only present in 19% of our sample consistent with other DS and ADAD reports [19, 24, 25, 64, 98] and occurred mainly in the absence of TDP-43 pathology, suggesting a dissociation between these pathologies possibly due to the younger age of individuals with DS compared to the aged population with HS and TDP-43.

*ARTAG:* Our preliminary findings suggest that ARTAG is present in 7% of adults with DS and was identified only in those with dementia. A frequency of ~ 20% has been reported in adults with DS over age 40 years in two independent autopsy cohorts. A higher frequency of ARTAG has been identified in over 30% of the population above 80 years of age [51] and is associated with LATE-NC and arteriolosclerosis or brain infarcts, which suggests that this pathology may also be associated with aging in DS. Importantly, ARTAG evaluation in our autopsy cohort was limited, as the available tau slides did not allow for systematic evaluation of this pathology across all suggested brain regions or full characterization of pathological subtypes. A detailed characterization of ARTAG, as well as other tau-related pathologies such as argyrophilic grain disease, will contribute greatly to the characterization of other tau-related pathologies in DS.

In summary, the co-pathologies observed in this study resemble those seen in aging and AD populations. The lower frequency of these pathologies in DS suggest they are primarily driven by age rather than by ADNC. While some preclinical evidence suggests that other genes in chromosome 21 can modulate amyloid deposition [79, 125], the contribution of chromosome 21 genes to the development of co-pathologies remains to be investigated. For example, the triplication of SOD1, S100B, DYRK1A, BACE-2, and SYNJ1 may contribute to the progression of AD neuropathology, oxidative damage, neuroinflammation, tau, or amyloid clearance, neuronal dysfunction, and potentially influence the development of co-pathologies and onset and progression of cognitive decline.

### Mixed neuropathologies

In our autopsy cohort, only a third (18/63) of all DS cases (with and without dementia) exhibited pure ADNC. This finding aligns with data from community and research based non-DS autopsy cohorts where only a small proportions of individuals with ADNC did not show additional co-pathologies [50, 100–102, 110, 115]. Most individuals in our cohort (71%) displayed ADNC and at least one or two co-pathologies, with CAA being the most frequent, followed by a combination of CAA and another neurodegenerative pathology (LP, LATE-NC, HS), a finding that aligns with other AD cohorts where vascular pathology, including CAA, is the most common co-pathology [50, 102, 115]. While the burden of co-pathologies may be lower than that reported in sporadic AD cohorts, this likely reflects the younger age of people with DS. Indeed, people with EOAD, who are usually younger, have a smaller number of co-pathologies than older individuals with LOAD [115] and a higher number have ADNC only or ADNC with CAA, similar to our DS cohort. Another interesting finding was the identification of 19 distinct pathological combinations across the DS cohort, suggesting that, despite being a genetically homogenous population, there is a significant degree of heterogeneity in the combination of co-pathologies. Further, pure ADNC was present in a higher proportion in those without dementia (50%) compared to those with dementia (25%), individuals with dementia carried more frequently three or more co-pathologies, and a greater heterogeneity of pathological combinations was observed in the dementia group. While these differences did not reach significance, possibly because we were limited by statistical power, such observations are important in light of findings reporting that co-pathologies have cumulative effects on cognitive performance and lower the threshold for cognitive decline in those with ADNC [50, 100, 101, 115]. Whether co-pathologies exert cumulative effects on cognition in DS, is an important question that requires investigation in larger autopsy cohorts with comprehensive longitudinal clinical and biomarker assessments.

## Limitations

The ADRC autopsy cohort includes volunteers, introducing a potential selection bias that may not represent the broader DS population. Compared to other neuropathology studies in the AD field, our small sample size limits investigating the impact of co-pathologies, demographic factors, and comorbidities on dementia in this cohort. We would like to emphasize that this autopsy cohort represents over 25 years of dedicated brain donation efforts through the UCI ADRC, UKY-ADRC and ABC-DS and represents the largest clinically and neuropathologically characterized DS brain donor cohort, making it a valuable and unique resource. In addition, the eight DS cases without dementia took over 25 years to identify and we believe that reporting these cases, even in small numbers, is an important scientific contribution. New brain donation efforts are underway, including the DS Biobank consortium and the INCLUDE Biorepository which will significantly expand the sample size in the coming years [2]. As DS brain donation programs linked to longitudinal assessments of people with DS like in ABC-DS continue to grow, we will be able to examine the relationship between ADNC and demographic variables and dementia status, the impact of co-pathologies on the onset and rate of cognitive decline, the impact of lifestyle factors and comorbidities cognitive on decline, the relationship between co-pathology burden and dementia severity, the association between *APOE* and co-pathologies, and the extent to which resilience and resistance mechanisms (neuropathological, genetic, lifestyle-related) protect against dementia in DS.

The limited number of brain sections sampled in routine neuropathological evaluations may result in underreporting some pathologies, particularly microinfarcts and hemorrhages; for this reason, we did not include these findings when accounting for co-pathologies. While moderate-to-severe atherosclerosis, arteriolosclerosis and CAA have been associated with cognitive impairment in the general population, the neuropathological thresholds at which these pathologies contribute to cognitive decline in DS remain unclear. We applied the same severity criteria used in the general population; however, given the distinct vascular biology of DS, these thresholds need to be confirmed in larger clinicopathological studies. Data on systematic vascular comorbidities were not available for this study, and their potential contribution to the cerebrovascular profile of our autopsy cohort could not be assessed. Our cohort includes one individual below age 50 years and one below age 53 years, who died before reaching the typical mean age of dementia onset in DS. We cannot ascertain whether these individuals, had they survived longer, may have eventually developed dementia. However, the presence of dementia in individuals under 50 years highlights the variability in age of dementia onset in DS. Our cohort predominantly represents a white population, highlighting the need for more diverse cohorts in DS research. Finally, although we used standardized and systematic guidelines, the scoring system remains semiquantitative. Quantitative digital pathology coupled with molecular profiling may provide more precise characterization of these cases, particularly those presenting atypical AD profiles. Such approaches may also render more accurate comparisons of pathological burden across individuals and identification of subtle differences that are not captured with semiquantitative approaches at the macro and microscopic levels.

## Conclusions

In conclusion, this study demonstrates that most adults with DS and ADNC over 40 years of age exhibit co-pathologies, with pure ADNC present in a minority of cases. CAA was the most frequent co-pathology, underscoring the cerebrovascular pathological burden in DS. This susceptibility to CAA has implications for the safety of amyloid-lowering immunotherapies as it may increase the risk of adverse vascular effects in response to these treatments. Despite being a homogenous population with genetically driven AD, there was significant heterogeneity in the pathological combinations observed in adults with DS, highlighting that people may develop distinct neuropathological profiles despite sharing the same AD genetic driver. Individuals with DS without dementia had larger brain weights and less frequent severe hippocampal atrophy and LC hypopigmentation and displayed intermediate ADNC. While non-significant, we note that LATE-NC and HS were not present in this group. These individuals may have had resilient and resistance mechanisms that protected them from cognitive decline.

## Supplementary Information

Below is the link to the electronic supplementary material.

Supplementary file1 (PDF 694 KB)

## Data Availability

The datasets used in this study are available from the corresponding author upon reasonable request at https://abc-ds.org/.

## Abbreviations

ABC-DS: Alzheimer’s Biomarker Consortium–Down Syndrome
Aβ: Amyloid-beta
AD: Alzheimer’s disease
ADAD: Autosomal dominant Alzheimer’s disease
ADNC: Alzheimer’s disease neuropathologic change
ADRC: Alzheimer’s Disease Research Center
APOE: Apolipoprotein E
CAA: Cerebral amyloid angiopathy
CERAD: Consortium to Establish a Registry for Alzheimer’s disease
DS: Down syndrome
EOAD: Early-onset Alzheimer’s disease
H&E: Hematoxylin and eosin
HS: Hippocampal sclerosis
ID: Intellectual disability
LATE-NC: Limbic-predominant age-related TDP-43 encephalopathy neuropathological change
LP: Lewy pathology
LOAD: Late-onset Alzheimer’s disease
NACC: National Alzheimer's Coordinating Center
NFT: Neurofibrillary tangle
NIA-AA: National Institute on Aging-Alzheimer’s Association
TDP-43: Transactive response DNA-binding protein 43 kDa
