# Supplementary material for: Frequency of mixed neuropathologies in individuals with Down syndrome with and without Alzheimer's dementia
Source: Acta Neuropathol. 2026 May 15;151(1):55. doi: 10.1007/s00401-026-03028-z (PMC13179220; doi:10.1007/s00401-026-03028-z)
Supplement: Supplementary file 1 — Supplementary file1 (PDF 694 KB) [file 401_2026_3028_MOESM1_ESM.pdf]

Supplementary Information

Frequency of mixed neuropathologies in individuals with Down syndrome with and without Alzheimer's dementia

Journal: Acta Neuropathologica

Lisi Flores-Aguilar[1], Thomas D. Zaikos[2], Isabel Rivera[1], Sierra T. Wright[1], Jerry Lou[1], Brianna Gawronski[3], Lourdes Gonzalez[3], Jillian V. Berry[1,4], Jeremy Rouanet[1], Natalie C. Edwards[5-7], Dan K. Hoang[3], Kevin Wood[3], Ann-Charlotte Granholm[8], Elliott J. Mufson[9], Edwin S. Monuki[1,10], Milos D. Ikonomovic[11-13], Julia Kofler[14], Eric W. Doran[15], Ira T. Lott[15], Minodora O Totoiu[15], Christy L. Hom[16], Florence Lai[17], William H. Yong[1], Frederick A. Schmitt[18], Jordan Harp[18], Peter T. Nelson[18-19], Jose Gutierrez[6], Patrick J. Lao[5-6], Donna M. Wilcock[20], Adam M. Brickman[5-6], Elizabeth Head[1]*, for the Alzheimer's Biomarker Consortium-Down syndrome (ABC-DS).

[1]- Department of Pathology and Laboratory Medicine, University of California Irvine, Irvine, CA, USA
[2]- Department of Microbiology and Molecular Genetics, University of California Irvine, Irvine, CA, USA
[3]- Institute for Memory Impairments and Neurological Disorders (MIND), University of California Irvine, Irvine, CA, USA
[4]- Department of Neurology, University of California Irvine, Irvine, CA, USA
[5]- Taub Institute for Research on Alzheimer's Disease and the Aging Brain, Columbia University, NY, USA
[6]- Department of Neurology, Vagelos College of Physicians and Surgeons, Columbia University, New York City, USA.
[7]- Department of Neuroscience, Columbia University, New York City, NY, USA.
[8]- Department of Neurosurgery, University of Colorado Anschutz Medical Center, Aurora, USA.
[9]- Department of Translational Neuroscience and Neurology, Barrow Neurological Institute, Phoenix, AZ, USA.
[10]- Sue and Bill Gross Stem Cell Center, University of California Irvine, Irvine, CA, USA
[11]- Department of Neurology, University of Pittsburgh, PA, USA
[12]- Department of Psychiatry, University of Pittsburgh, PA, USA
[13]- Geriatric Research Education and Clinical Center, VA Pittsburgh HS, Pittsburgh, PA, USA
[14]- Department of Pathology, University of Pittsburgh, PA, USA
[15]- Department of Pediatrics, University of California Irvine, Orange, CA, USA.
[16]- Department of Psychiatry and Human Behavior, University of California Irvine, Irvine, CA, USA.
[17]. Department of Neurology, Harvard University, MA, USA
[18]- Department of Neurology, Sanders-Brown Center on Aging, University of Kentucky, Lexington
[19]- Department of Pathology and Laboratory Medicine, Division of Neuropathology, University of Kentucky, Lexington, Kentucky, USA.
[20]- Department of Neurology, Indiana University, IN, USA

***Corresponding author:**

Dr. Elizabeth Head

Email: heade@uci.edu

Supplemental Figures

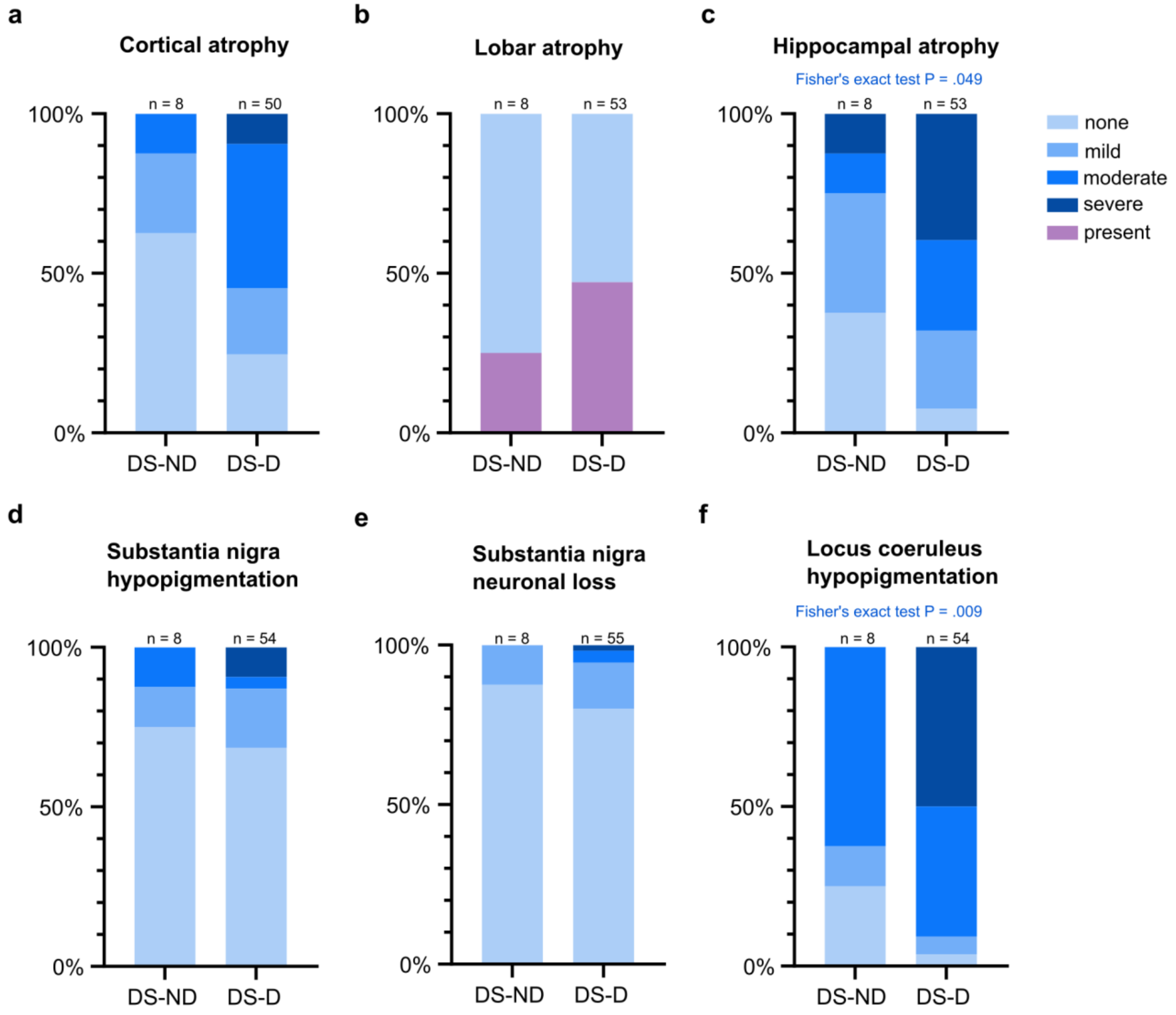

Supplemental Fig. 1 Gross neuropathological findings stratified by dementia status. **a-f** Stacked bar charts illustrating the distribution of gross neuropathological findings in individuals with DS with and without dementia. The frequency of hippocampal atrophy severity and locus coeruleus hypopigmentation was greater in those with dementia **(c,d)**. No significant differences were observed between groups for the other findings. DS-ND = DS without dementia; DS-D = DS with dementia

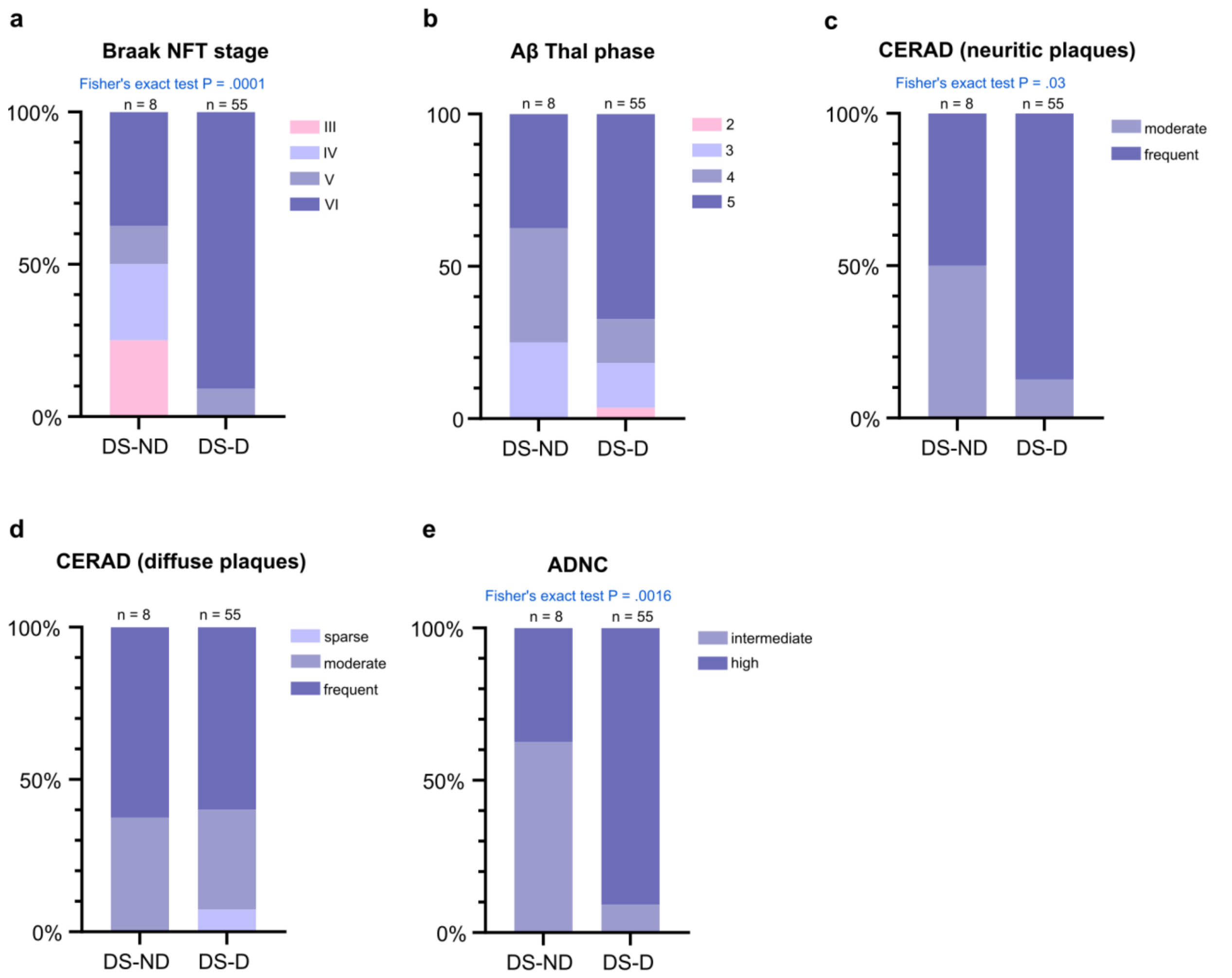

Supplemental Fig. 2 Alzheimer's disease neuropathological change stratified by dementia status. **a-e** Stacked bar charts illustrating the distribution of ADNC in individuals with DS with and without dementia. Braak NFT stage distribution was more advanced in individuals with dementia (a). CERAD neuritic plaques were more frequent in those with dementia (c). High ADNC was more frequent in individuals with dementia. No significant differences were observed for Thal phase or diffuse plaque scores (b,d). DS-ND = DS without dementia; DS-D = DS with dementia; ADNC = Alzheimer's disease neuropathological change; CERAD = Consortium to Establish a Registry for Alzheimer's disease; NFT = neurofibrillary tangles, Aβ = amyloid-beta

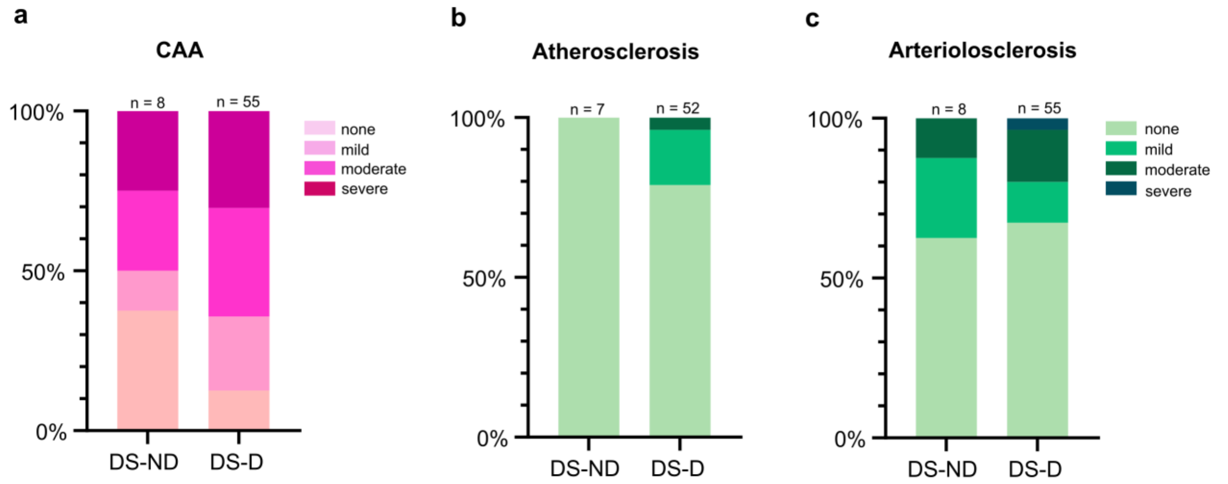

Supplemental Fig. 3 Cerebrovascular pathology stratified by dementia status. **a-c** Stacked bar charts illustrating the distribution of CAA, atherosclerosis and arteriolosclerosis in individuals with DS with and without dementia. **a** Moderate to severe CAA was present in most individuals regardless of dementia status. **b** Atherosclerosis was absent in individuals without dementia and predominantly mild forms were present in a minority of individuals with dementia. **c** Arteriosclerosis was also infrequent in both groups. DS-ND = DS without dementia; DS-D = DS with dementia; CAA = cerebral amyloid angiopathy

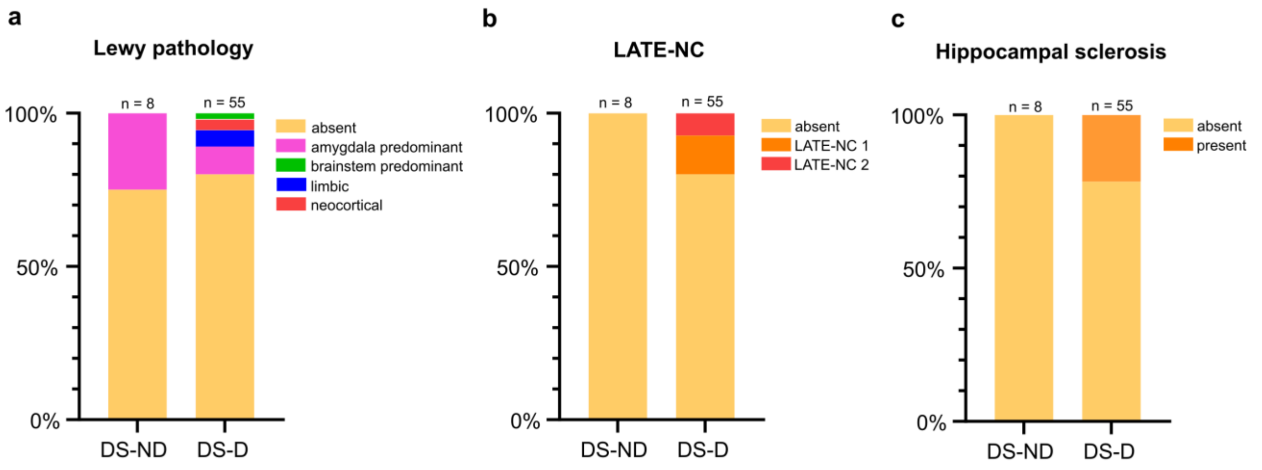

Supplemental Fig. 4 Lewy pathology, LATE-NC, and hippocampal sclerosis stratified by dementia status. **a-c** Stacked bar charts illustrating the distribution of Lewy pathology, LATE-NC, and hippocampal sclerosis in individuals with DS with and without dementia. **a** Lewy pathology was present in a similar frequency in both groups, with amygdala-predominant being the most frequent category. **b** LATE-NC was absent in individuals without dementia and present in 20% of individuals with dementia, with most being at stage 1. **c** HS was also absent in individuals without dementia and present in 22% of those with dementia. DS-ND = DS without dementia; DS-D = DS with dementia; LATE-NC = limbic predominant age-related TDP-43 encephalopathy neuropathological change

a

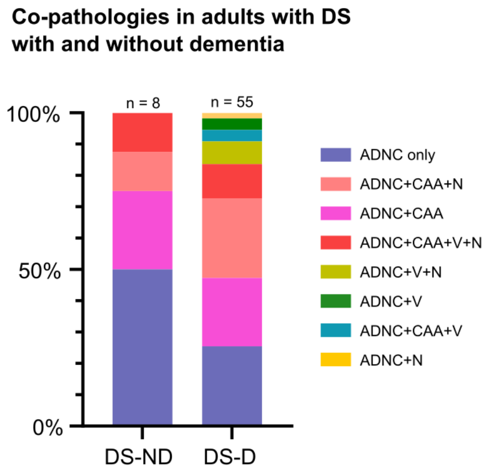

b

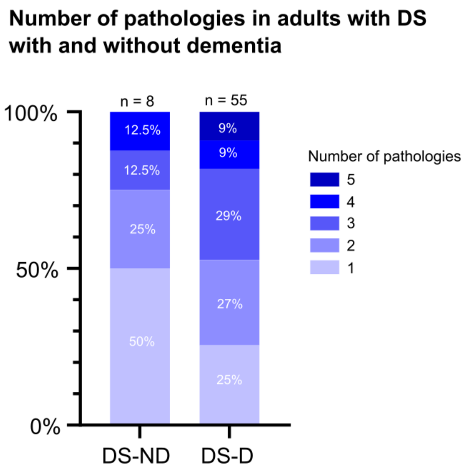

c

Pathological combinations in DS without dementia

# cases	Frequency	Pathological combinations
4	50%	ADNC
2	25%	ADNC + CAA
1	13%	ADNC + CAA + LP
1	13%	ADNC + CAA + V + LP

n = 8

Pathological combinations in DS with dementia

# cases	Frequency	Pathological combinations
14	25%	ADNC
13	24%	ADNC + CAA
5	9%	ADNC + CAA + HS
3	5%	ADNC + CAA + LP
3	5%	ADNC + CAA + V
3	5%	ADNC + CAA + LATE-NC
2	4%	ADNC + CAA + V + LP + HS
1	2%	ADNC + CAA + V + LP
1	2%	ADNC + V
1	2%	ADNC + LATE-NC
1	2%	ADNC + V + HS
1	2%	ADNC + V + LATE-NC
1	2%	ADNC + V + LP + LATE-NC
1	2%	ADNC + CAA + LP + HS
1	2%	ADNC + CAA + LP + LATE-NC
1	2%	ADNC + V + LATE-NC+ HS
1	2%	ADNC + CAA + LP + HS+ LATE-NC
1	2%	ADNC + CAA +V + LP + LATE-NC
1	2%	ADNC + CAA +V + LATE-NC + HS

n = 55

Supplemental Fig. 5 Mixed neuropathologies in adults with Down syndrome stratified by dementia status. **a** Stacked bar charts illustrating the distribution of co-pathologies in individuals with and without dementia. Cases were classified into eight groups based on the presence of co-pathologies: ADNC only, CAA (moderate or severe), vascular pathology (V; moderate or severe atherosclerosis or ateriolosclerosis), and other neuropathologies (N; Lewy pathology, LATE-NC, and/or hippocampal sclerosis). **b** Stacked bar chart illustrating the distribution of the total number of individual co-pathologies (ADNC, CAA, V, atherosclerosis/arteriosclerosis, Lewy body pathology, LATE-NC, hippocampal sclerosis) in individuals with and without dementia. Most individuals without dementia carried one to two pathologies, while one to three pathologies were more frequent in those with dementia **c** Table illustrating the pathological combinations identified in individuals with and without dementia. Notice the heterogeneity of the neuropathological burden in the group with dementia. DS-ND = DS without dementia; DS-D = DS with dementia; ADNC = Alzheimer's disease neuropathological change, CAA = cerebral amyloid angiopathy, LP = Lewy pathology, HS = hippocampal sclerosis, LATE-NC = limbic predominant age-related TDP-43 encephalopathy neuropathological change

Supplemental Tables

Supplemental Table 1. Association between APOE ε4 and CAA severity in adults with Down syndrome

	**none/mild CAA** n (%)	**moderate-severe CAA** n (%)	**P value**
**APOE ε4 -**	18 (45)	22 (55)	.27
**APOE ε4 +**	6 (29)	15 (71)	

APOE = Apolipoprotein E, CAA=cerebral amyloid angiopathy

Supplemental Table 2. Vessel mineralization in adults with Down syndrome

	All DS n (%)	DS without dementia n (%)	DS with dementia n (%)	P value
**Vessel mineralization**	18	4	14	
Present	15 (83)	2 (25)	13 (93)	.11
Absent	3 (17)	2 (50)	1 (7)	

Supplemental Table 3. Frequency of ARTAG in individuals with DS

	**All DS** n (%)	**DS without dementia** n (%)	**DS with dementia** n (%)	**P value**
**ARTAG** (n)	57	8	49	
Present	4 (7)	0	4 (8)	>.99
Absent	53 (93)	8 (100)	45 (92)	

The first column presents findings for the full DS autopsy cohort (All DS). The following columns present findings stratified by dementia status and group comparisons. ARTAG = Aging-related tau astrogliopathy, DS= Down syndrome
